# Mussel-Inspired Multifunctional Polyethylene Glycol Nanoparticle Interfaces

**DOI:** 10.3390/biomimetics9090531

**Published:** 2024-09-04

**Authors:** Carolina Casagualda, Alba López-Moral, Paula Alfonso-Triguero, Julia Lorenzo, Ramon Alibés, Félix Busqué, Daniel Ruiz-Molina

**Affiliations:** 1 Catalan Institute of Nanoscience and Nanotechnology (ICN2), CSIC, and The Barcelona Institute of Science and Technology (BIST), Campus UAB, Bellaterra, 08193 Barcelona, Spain; 2 Departament de Química, Universitat Autònoma de Barcelona, Bellaterra, 08193 Barcelona, Spain; 3 Institut de Biotecnologia i de Biomedicina and Departament de Bioquímica i Biologia Molecular, Universitat Autónoma de Barcelona, Bellaterra, 08193 Barcelona, Spainjulia.lorenzo@uab.cat (J.L.); 4 Centro de Investigación Biomédica en Red (CIBER), Bioingeniería, Biomateriales y Nanomedicina, 08193 Cerdanyola del Vallès, Spain

**Keywords:** catechol, functional coatings, bioinspired, functional nanoparticles, biocompatible, magnetite NPs, MSNPs

## Abstract

Nanoparticles (NPs) are receiving increasing interest in biomedical applications. However, due to their large surface area, in physiological environments, they tend to interact with plasma proteins, inducing their agglomeration and ultimately resulting in a substantial efficiency decrease in diagnostic and therapeutic applications. To overcome such problems, NPs are typically coated with a layer of hydrophilic and biocompatible polymers, such as PEG chains. However, few examples exist in which this property could be systematically fine-tuned and combined with added properties, such as emission. Herein, we report a novel mussel-inspired catechol-based strategy to obtain biocompatible and multifunctional coatings, using a previously developed polymerization methodology based on the formation of disulfide bridges under mild oxidative conditions. Two families of NPs were selected as the proof of concept: mesoporous silica NPs (MSNPs), due to their stability and known applications, and magnetite NPs (Fe_3_O_4_ NPs), due to their small size (<10 nm) and magnetic properties. The PEG coating confers biocompatibility on the NPs and can be further functionalized with bioactive molecules, such as glucose units, through the end carboxylic acid moieties. Once we demonstrated the feasibility of our approach to obtaining PEG-based coatings on different families of NPs, we also obtained multifunctional coatings by incorporating fluorescein functionalities. The resulting coatings not only confer biocompatibility and excellent cell internalization, but also allow for the imaging and tracking of NPs within cells.

## 1. Introduction

The substantial surface area of nanoparticles (NPs) makes them prone to interacting with plasma proteins in physiological environments, leading to agglomeration and precipitation. NPs are also considered intruders by the innate immunity system and engulfed by macrophage cells. In both scenarios, the particles are removed from the blood circulation system and lose their function quickly, leading to a dramatic diagnosis and therapy efficiency reduction. To address these issues, NPs are usually coated with a layer of hydrophilic and biocompatible polymers, such as dextran [1], dendrimers [2], or mainly PEG chains [3]. In this last case, several approaches have been followed to ensure proper surface functionalization. Undoubtedly, one of their most common uses is their covalent attachment to catechol units, drawing inspiration from numerous marine sandcastle worms, barnacles, or mussels. These organisms firmly attach to underwater surfaces, preventing displacement by currents and tides while carrying out essential life functions. Their remarkable adhesive capabilities stem from catechol-based molecules, serving as a foundational element in the creation of innovative synthetic coatings in many different laboratories worldwide.

**Figure 1:**
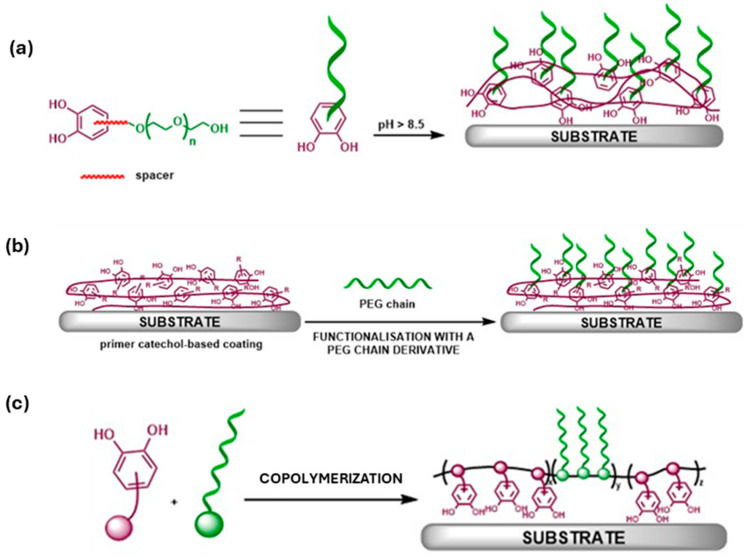
Overview of strategies followed in the literature using catechol-based polyethylene glycol (PEG) coatings to endow nanoparticles (NPs) with colloidal stability and biocompatible properties in physiological media. (**a**) Polymerization of cat-PEG-based molecules, where catechol and PEG chains are covalently bonded or with a short spacer in between. Surfaces are coated via ex-situ treatments. (**b**) Preliminary coating of the surface with catechol-based polymers, commonly PDA, and further functionalization with PEG derivatives through covalent bonds. (**c**) Copolymerization through reactive pendant groups between molecules containing catechol moieties and PEG chains.

Indeed, so far, numerous research examples have showcased the use of catechol-based polyethylene glycol (cat-PEG) coatings to confer nanoparticles (NPs) with colloidal stability, antifouling and antimicrobial properties, as well as biocompatibility and biodegradability in physiological environments. For this purpose, one of the main approaches used is the polymerization of molecules covalently linking catechol and PEG chains, either with or without a short spacer in between. The polymerization occurs under oxidative conditions at basic pH, and surface coating is achieved through ex situ treatments (Figure 1a). Following this approach, numerous metallic, inorganic, and polymeric surfaces have been coated with polyethylene glycol dopamine (PEG-DA) polymers [4,5]. For instance, examples of coated Fe_3_O_4_, MnO, and Au NPs dispersed in both aqueous and organic environments have been reported [6], with those with plasmon resonance responses (Au and Ag NPs) being suitable for diagnostic and therapy applications [7], or reduced graphene oxide (rGO) coated with cat-PEG polymers for the controlled release of doxorubicin (DOX) [8]. Overall, with this polymerization strategy, polymers [9,10,11], metals [12,13,14], metal oxides [15,16,17,18], and electrospun nanofiber meshes [19], reduced graphene oxide [20], and glass and polystyrene beads [21], ceria nanoparticles [22], or iron oxide nanocubes (IONCs) [23], have been effectively coated.

A second alternative synthetic methodology involves first the formation of a catechol-based coating, typically polydopamine (PDA), followed by functionalization with PEG chain derivatives via covalent bonds (Figure 1b). Following this methodology, several representative examples have been reported, including, for instance, MoS_2_ nanosheets for drug delivery and photothermal cancer treatment [24], PEGylated carbon nanotubes (CNTs) for the intracellular delivery of doxorubicin [25], multi-walled carbon nanotubes (MWCNT) [26], and Fe_3_O_4_ NPs [27] or catechol-conjugated hydrogels produced by thiourea-quinone coupling [28].

All in all, numerous examples of cat-PEG coatings have already been described in the literature. However, the potential for obtaining multifunctional coatings is less frequently explored despite its interest, bringing the possibility of combining the advantages of PEG groups with, for instance, fluorescent tags for imaging. For this purpose, we hypothesize that the copolymerization of catechol moieties bearing PEG chains and other different functional groups represents one of the best scenarios (Figure 1c). With this third approach, the functionalization of polyether copolymers [29], coating Fe_3_O_4_ NPs for bioimaging [30]/gene therapy [31]/contrast agents [32], SiO_2_ NPs with cisplatin-controlled release kinetics [33], AuNPs as smart carriers for the doxorubicin drug [34], and polymeric micelles [35,36] or nanocarriers [37] as drug delivery systems have already been reported. Further examples involve multidentate cat-PEG-coated magnetic nanocrystals [38] or NPs [39], the surface modification of CNTs [40], metal–phenolic networks that reduce the cellular oxidative stress [41], or increasing the binding affinity of Au@PDA NPs [42]. Nevertheless, despite these numerous examples, no examples of fine-tuning added properties have been reported so far, as far as we know. Therefore, there is a strong need for the development of novel synthetic approaches to address this limitation.

**Figure 2:**
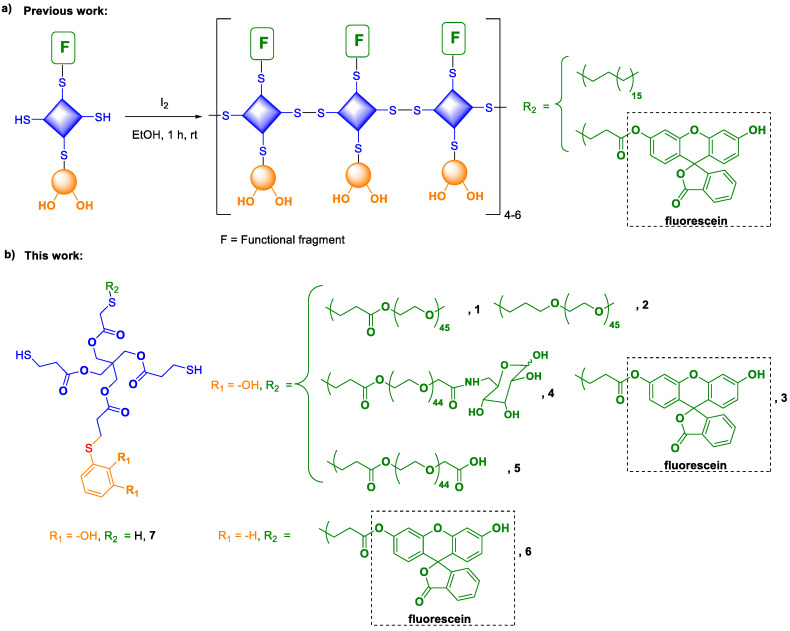
(**a**) Oligomeric materials previously obtained by our research group [43] by formation of disulfide bridges between functionalized catechols using iodine as oxidant. (**b**) Functionalized catechols, some of which include a PEG chain, used in this work to obtain new functional or multifunctional coatings.

We recently reported a novel family of colorless coatings that relies on a catechol-grafted polymeric architecture linked through disulfide bridges [43], being the basic framework for the modular design of all monomers, including the pentaerythritol tetrakis(3-mercaptopropionate). This scaffold is linked to the catechol unit through the addition of one of the thiol groups to the oxidized *o*-quinone form. A second thiol group allows for the simultaneous incorporation of a functional group bearing a terminal vinyl or acrylate group using a thiol-ene click reaction. Lastly, the remaining two accessible thiols undergo polymerization through the formation of disulfide bridges, under mild and selective conditions, using iodine (Figure 2a). Pursuing this approximation, herein, we report new multifunctional coatings combining methyl-, glucose- or carboxylic acid-caped polyethylene glycol chains (compounds **1** and **2**, and **4** and **5**, respectively) with fluorescein fragments (**3**) (see Figure 2b). As representative supports, two families of NPs were chosen: (I) mesoporous silica NPs (MSNPs), selected for their stability and ability to encapsulate and release drugs, and (II) small (<10 nm) magnetic NPs (MNPs, Fe_3_O_4_ NPs), which are relevant in magnetic resonance imaging (MRI) [44], drug and gene delivery [45], magnetothermal therapy [46], biosensors and bioseparation [47]. Finally, and for comparison purposes, we also evaluated related model coatings bearing styrene moieties instead of catechols, to assess their role in the adhesion and coating capabilities.

## 2. Materials and Methods

### 2.1. General Procedures

The solvents, chemicals, and reagents were acquired with high quality without any need for further purification from various commercial chemical companies, such as Merck (Darmstadt, Germany), Scharlab (Sentmenat, Spain), Apollo Scientific (Cheshire, UK), Alfa Aesar (Kandel, Germany), and TCI (Zwijndrecht, Belgium). All reactions were monitored by analytical thin-layer chromatography (TLC) using silica gel 60 pre-coated aluminum plates (0.20 mm thickness). Flash column chromatography was performed using silica gel Geduran^®^ SI 60 (40–63 µm). ^1^H NMR and ^13^C NMR spectra were recorded at 298 K at 250, 360, 400 MHz, and 90, 100 MHz, respectively. Proton chemical shifts are reported in ppm (δ) (CDCl_3_, δ 7.26 or CD_3_COCD_3_, δ 2.06 or CD_2_Cl_2_ δ 5.32). Carbon chemical shifts are reported in ppm (δ) (CDCl_3_, δ 77.16 or CD_3_COCD_3_, δ 29.8, and CD_2_Cl_2_ δ 53.5. Infrared spectra (IR) were recorded on a Bruker Tensor 27 Spectrophotometer equipped with a Golden Gate Single Refraction Diamond ATR (attenuated total reflectance) accessory. Peaks are reported in cm^−1^. HRMS were recorded in an Agilent 6454 Q-TP spectrometer with an Agilent Jetstream Technology (AJT) source using electrospray ionization (ESI) or electronic impact (EI). The molecular weight distribution determined by gel permeation chromatography (GPC) was made using an Agilent Technologies 1260 Infinity chromatograph and THF as a solvent. The instrument is equipped with three gel columns: PLgel 5 μm Guard/50 × 7.5 mm^2^, PLgel 5 μm 10,000 Å MW 4 K−400 K, and PL Mixed gel C 5 μm MW 200−3 M. Calibration was made by using polystyrene standards. In each experiment, the freshly prepared polymer sample of interest was dissolved in THF (1–2 mg/mL) and immediately analyzed by GPC (1 mL/min flow; 30 °C column temperature). Transmission electron microscopy (TEM) analyses were performed with a JEOL JEM-1400 transmission microscope operating at 120 kV. TEM samples were prepared by dipping a carbon copper grid into a dilute suspension of the particles in freshly sonicated hexane. The average particle size and its standard deviation were estimated by measuring the edge length of at least 200 particles using the software ImageJ (Fiji). Data were fitted to a log-normal function and the polydispersity index (PDI) was calculated.

### 2.2. Synthesis of Monomers

The synthesis of all new monomers considered and their full chemical characterization are described in the literature [43] (compounds **3** and **6**) or in the supporting information (compounds **1**, **2**, **4**, and **5**).

### 2.3. Synthesis and Characterization of Oligomeric Materials ***P1***, ***P2***, ***P5***, and ***C2***–***3***

As a general procedure, a solution of 35 mM of resublimed iodine in EtOH 96% (1.1 equiv.) was added dropwise to a ~7 mM solution of CATPEG bis-thiol **1**, **2** or **5** (or a 4:1 mixture of **2** and **3**) in EtOH 96%, containing an additional 0.035 equivalents of related cat-Me-(SH)_2_ **11** as doping reagent to improve crosslinking. The reaction mixture was stirred for 1 h at rt, after which a yellowish solid precipitated. The supernatant was decanted, and the solid was washed with fresh EtOH 96% three times and dried under vacuum, yielding 45%, 28%, 55%, and 48% of oligomeric material **P1**, **P2**, **P5**, and **C2**–**3**, respectively.

The products obtained from the polymerization reaction were characterized by different techniques, such as ^1^H NMR and gel permeation chromatography (GPC). The ^1^H NMR experiments were performed in THF-d_8_, whereas GPC analyses were performed in THF. In the ^1^H NMR, the disappearance of the thiol’s peak of monomers was observed around 1.6 ppm, thus indicating that the disulfide bonds had been formed. To prepare GPC samples, the derivatives obtained after polymerization were dissolved in THF (1 or 2 mg/mL) and filtered through 0.22 µm nylon filters.

### 2.4. Coating of Mesoporous Silica Nanoparticles

As a general procedure, 10 mg of NPs (synthesized as described in *Chemistry–A European Journal* 2014, 20 (47), 15443–15450) were dispersed in 1 mL HPLC-grade CH_2_Cl_2_, a solution of ~7 mM of the corresponding oligomer was added, and the mixture was left to stir at 500 rpm overnight at rt. The final dispersion was centrifuged at 14,000 rpm for 2 min, and the resulting MSNPs were washed with fresh CH_2_Cl_2_ three times to remove the excess of mPEG derivatives.

### 2.5. Coating of Magnetite Nanoparticles

#### 2.5.1. Preparation of Magnetite NPs (MNPs) [48]

Fe(acac)_3_ (0.441 g, 1.24 mmol), sodium oleate (0.213 g, 0.68 mmol), and oleic acid (1.485 g, 5.26 mmol) in a mixture of solvents composed of dibenzyl ether (10 mL), 1-octadecene (10 mL), and 1-tetradecene (3 mL) were heated up to 110 °C and kept at this temperature during 1.5 h under vacuum. During this time, reactive intermediates were formed. The reaction mixture was then heated up to 290 °C with a heating rate of 3 °C/min, and kept at this temperature for 1 h under Ar flow. In this step, nucleation and the subsequent growth of the NPs took place. Finally, the reaction mixture was cooled down to 50 °C. To purify the MNPs, isopropanol (40 mL) and acetone (40 mL) were added to the reaction mixture, which was centrifuged (10,000 rpm, 12 min) to separate the MNPs from the supernatant. The MNPs were resuspended in a mixture of CHCl_3_ (10 mL), acetone (40 mL), and isopropanol (40 mL), and the same centrifugation conditions were applied. Finally, the MNPs were redispersed in CHCl_3_ (~6.5 mg/mL).

#### 2.5.2. Coating of MNPs: Synthesis of Fe_3_O_4_@**P5** NPs

To a suspension of MNPs (6.5 mg/mL, 2.6 mL, 17 mg), a solution of **P5** (10 mg, 0.004 mmol) in HPLC-grade CHCl_3_ (1.1 mL) was added. The reaction mixture was left to stir at rt for 3–4 days. The final MNPs were transferred to the aqueous phase and washed with CHCl_3_ once, and the water removed under vacuum.

#### 2.5.3. Functionalization of Fe_3_O_4_@**P5** NPs: Synthesis of Targeted Fe_3_O_4_@**P5**-Glucose NPs

To a suspension of Fe_3_O_4_@**P5** NPs (12 mg) cooled down to 0 °C, a solution of amino-glucopyranose **15** (15.4 mg, 0.09 mmol) in diisopropylethyl amine (DIPEA) (314 mL, 1.8 mmol) was added. After 10 min of stirring at 0 °C, 1-ethyl-3-(3-dimethylaminopropyl)carbodiimide (EDCI) (0.12 mmol) was added and the reaction mixture was stirred at rt for 3–4 days under Ar atmosphere. The final MNPs were transferred to the aqueous phase and washed with CHCl_3_ once, and the solvent was removed under vacuum. The resulting nanoparticles were treated with DCl 35% *w*/*w* in H_2_O and analyzed by ^1^H NMR.

In the case of working with protected amino-glucofuranose derivative **8**, the procedure followed was the same, but sugar derivative **8** along with 4-(dimethylaminopropoyl)pyridine (0.3 equivalents) was directly added into MNPs@**P7** suspension.

In the case of the partial deprotection of Fe_3_O_4_@**P5**-ProtecAminoFuranose NPs, to a ~1.5 mg/mL suspension of Fe_3_O_4_@**P5**-ProtecAminoFuranose NPs, a mixture of AcOH/H_2_O with the corresponding ratio was added, and the reaction mixture was stirred. Subsequently, the aqueous phase was washed with CHCl_3_ (x3) to remove non-polar impurities and evaporated under vacuum. The resulting NPs were treated with DCl 35% *w*/*w* in H_2_O and analyzed by ^1^H NMR.

### 2.6. Cytotoxicity and Internalization Assays of ***C2***–***3*** Coated MSNPs

#### 2.6.1. Cytotoxicity Assay

Human SH-SY5Y cells were cultured in DMEM/F-12 culture medium supplemented with 10% FBS and 1% antibiotic-antimycotic solution (Gibco-BRL, Carlsbad, CA, USA) and incubated at 37 °C in a humidified atmosphere with 5% CO_2_. Cells were seeded into a 96-well plate at a cell density of 3.0 × 10^3^ cells per well and then incubated for 24 h. **C2**–**3**-coated MSNPs were resuspended in the medium discussed above to achieve the different concentrations used in this assay: 0, 1, 5, 10, 25, 50, 100, and 200 μg/mL. Next, the cells were incubated at 37 °C, in a humidified atmosphere with 5% CO_2_ and 98% humidity for 24 h. The cytotoxicity effect was measured after 24 h treatment employing the PrestoBlue cell viability reagent (ThermoFisher, Waltham, MA, USA). PrestoBlue (10 μL; resazurin-based solution) was added to each well. After a two-hour incubation period (37 °C, 5% CO_2_, 98% humidity), the fluorescence was quantified using a fluorescent multilabel plate reader (Victor3, PerkinElmer, Waltham, MA, USA) with excitation at λ = 531 nm and recorded at λ = 572 nm. Cell cytotoxicity was evaluated in terms of cell viability and expressed as a percentage of the control conditions. Each experiment was repeated at least three times, and each concentration was tested in at least three replicates.

#### 2.6.2. Internalization Assay

Human SH-SY5Y cells were cultured as in the previous assay for 24 h. **C2**–**3**-coated MSNPs (200 μg/mL) were added to four of the six wells (the other two were used as a negative control). One of the wells with **C6–8**-coated MSNPs was washed with PBS, and its cells were fixed with 4% paraformaldehyde (PFA) for 15 min at rt. The PFA was then removed, and PBS was added to the well. This procedure was repeated at 3, 6, and 24 h with one of the wells with **C2**–**3**-coated MSNPs. The same procedure was performed at 6 and 24 h for one of the wells without **C2**–**3**-coated MSNPs. Subsequently, the samples were blocked by adding a blocking buffer (5% bovine serum albumin solution in PBS buffer supplemented with 0.1% Triton X-100) (1 mL) and incubated for 1 h at rt. The blocking buffer was then removed, and the samples were washed with PBS for 5 min in agitation. Next, the samples were incubated with 4′,6-diamidino-2-phenylindole (DAPI; 1/500) for 10 min at rt. After removing DAPI and washing the samples with PBS for 5 min in darkness, the samples were mounted on a drop of ProLong Gold.

## 3. Results and Discussion

### 3.1. Synthesis of Monomers ***1***–***6*** and the Corresponding Oligomeric Materials ***P1***–***5***

**Scheme 1:**
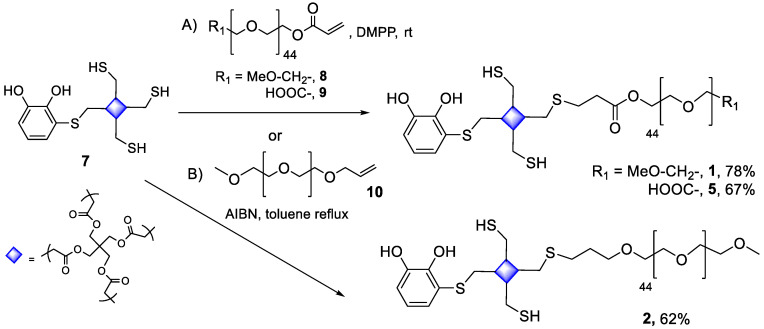

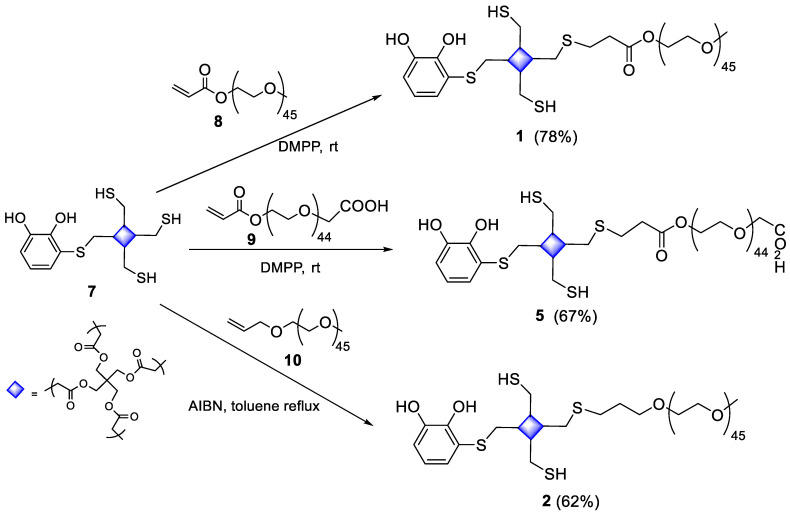
Synthesis of cat-PEG monomers **1**, **5**, and **2**.

Compounds **3** and **6**, intermediate **7**, and the corresponding oligomers **P3** and **P6** were synthesized in our previous work [43]. Cat-PEG derivatives **1**, **2**, and **5** were obtained following the same synthetic methodology (Scheme 1). Thus, the intermediate *S*-catechol tris-thiol **7** was coupled with heterobifunctional PEG derivatives **8**, **9**, and **10**, to render the corresponding cat-PEG monomers **1**, **5**, and **2**, in 78%, 67%, and 62% yields, respectively. The obtention of **1** and **5** was based on a radical catalyzed thiol-ene reaction between **7** and **8** or **9**, and the synthesis of **2** consisted of a thia-Michael reaction between **7** and **10** using dimethylphenyl phosphine as the catalyst. Conversely, compound **4**, which contains a glucose unit, could not be isolated due to the instability of its immediate precursor (Scheme S1 in the Supplementary Material).

**Scheme 2:**
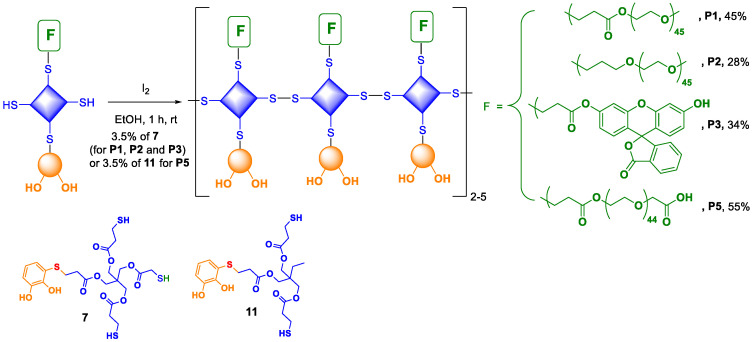
Synthesis of oligomeric materials **P1**, **P2**, **P3**, and **P5** from the corresponding monomers.

Next, we proceeded with the polymerization of the synthesized cat-PEG monomers **1**, **2**, and **5** under the same reaction conditions as in our previous work. Thus, oligomeric materials **P1**, **P2**, **P3**, and **P5** were obtained (Scheme 2) in 45%, 28%, 34%, and 55% yields, respectively, using iodine as an oxidant in EtOH. For **P1**, **P2**, and **P3**, 3.5% of the related cat-(SH)_3_ **7** as the doping reagent was used to enhance crosslinking. These materials, insoluble in EtOH but soluble in CHCl_3_, exhibited ^1^H NMR and IR spectra (see Supplementary Material) consistent with what was expected based on the precursors. In the ^1^H NMR spectrum, a decrease in the intensity of the thiol proton around 1.6 ppm was observed, as expected after the formation of disulfide bonds. According to the diffusion-ordered-spectroscopy (DOSY) NMR experiments, all the materials resulted in oligomers between 2 and 3 units. In the case of material **P2**, characterization was additionally performed using the GPC technique with THF as the solvent, revealing that it consisted of an oligomer ranging between 2 and 5 units. For materials **P1** and **P3**, GPC analyses were not feasible due to their insolubility in THF. Regarding the oligomeric material **P5**, 3.5% of the related cat-Me-(SH)_2_ **11** was used as the doping reagent to enhance crosslinking. The GPC analysis of the THF-soluble part of **P5** did not show any relevant peaks in the spectrum. Conversely, the corresponding precursor **5** displayed a distinct peak indicative of the monomeric compound, indirectly suggesting that monomer **5** was absent from the **P5** material.

### 3.2. Coating of Mesoporous Silica Nanoparticles

**Figure 3:**
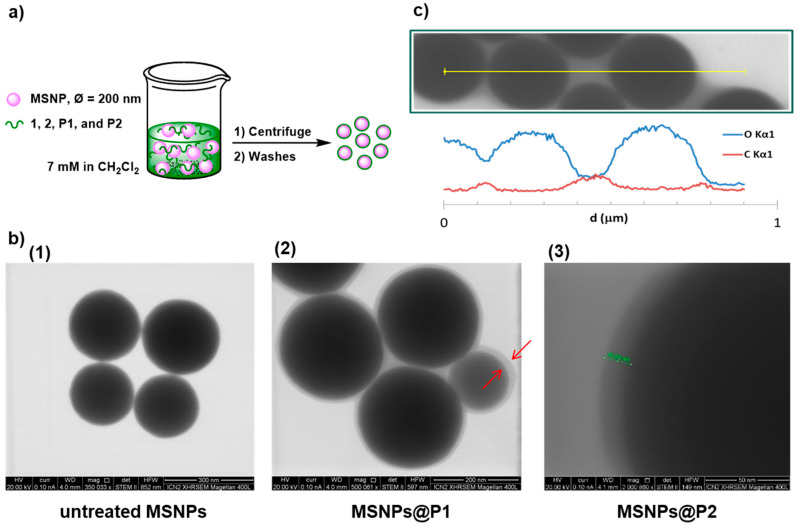

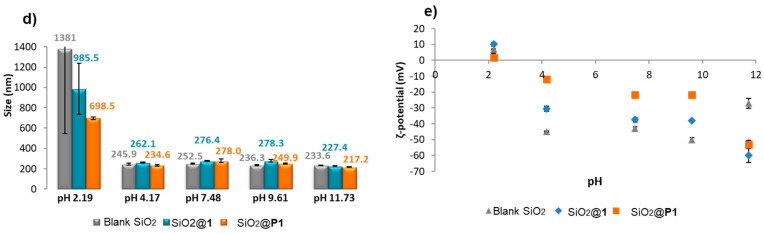
(**a**) Schematic representation of the procedure for coating onto MSNPs. (**b**) STEM images of untreated MSNPs (1), **P1**-coated MSNPS (2), and **P2**-coated MSNPS (3). (**c**) EDX line scan profile along the line drawn across the diameters of **P2**-coated MSNPs. (**d**) DLS analyses of untreated (gray) and treated (blue and orange) MSNPs at different pH. DLS values are the average of three measurements. (**e**) ζ-potential values of untreated (gray) and treated (blue and orange) MSNPs at different pH.

Mesoporous SiO_2_ NPs (MSNPs, Ø~200 nm) were dispersed in a ~7 mM CH_2_Cl_2_ solution of oligomers **P1** and **P2** and the corresponding monomers **1** and **2**, for comparison (Figure 3a). The mixture was then stirred overnight at rt and subsequently centrifuged at 14,000 rpm. The resulting MSNPs were washed with fresh HPLC-grade CH_2_Cl_2_ and characterized by different techniques, including scanning transmission electron microscopy (STEM), energy-dispersive X-ray spectroscopy (EDX), dynamic light scattering (DLS), and ζ-potential measurements. The results are shown in Figure 3.

**Figure 4:**
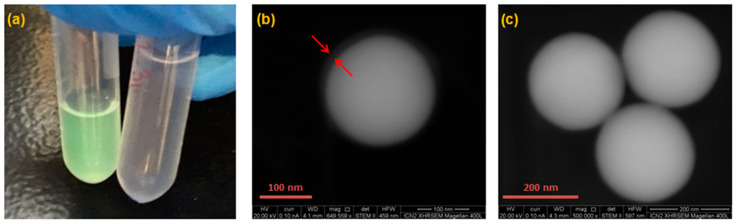
(**a**) **P3**-coated MSNPs (**left**) and **P6**-coated MSNPs (**right**) under UV lamp at 254 nm. STEM images of (**b**) MSNP@**P3**, where a coating layer can be identified and (**c**) MSNP@**P6**.

From the STEM images, the PEGylated coating can be distinguished as thin lighter layers (ca. 20 nm) surrounding the NPs, whereas the untreated MSNPs did not exhibit any organic shells (Figure 3b). In the case of MSNPs@**P2**, the coating was confirmed in the EDX line scan (Figure 3c), with carbon (red line) across the whole diameter of the coated particles, while the oxygen signal (blue line) remained circumscribed to their inner sections, i.e., to the mineral core. Pristine and coated MSNPs (0.5–1 mg) were also dispersed in 1 mL of aqueous solution at different pHs and analyzed by DLS and ζ-potential measurements. While both families of NPs were stabilized with sizes around 200–250 nm within the pH range of 4–12, significant ζ-potential differences were found (Figure 3e), qualitatively evidencing the effectiveness of the coating. In any case, at pH 2, aggregates larger than 1 µm were formed, which was attributed to the worse electrostatic stabilization and the gradual coating disaggregation from the NPs. The coating experiments were repeated under the same experimental conditions, now using **3** and **P3**. The STEM images showed a coating layer surrounding the NPs (Figure 4b, left) associated with bulk fluorescence under UV light (Figure 4a, left) or an optical microscope using the fluorescence mode and an Alexa Fluor 488 filter. Moreover, to prove the role of catechol units in the adhesive properties, the experiments were repeated using **6** and its corresponding oligomer, **P6**. The NPs treated with these derivatives did not exhibit any fluorescence (Figure 4a, right), and the STEM images revealed that MSNPs@**P6** resembled pristine MSNPs, devoid of any organic shells (Figure 4c). Thus, this simple example underscores the significant role of the catechol moiety as an anchor unit.

### 3.3. Coating of Fe_3_O_4_ NPs

**Figure 5:**
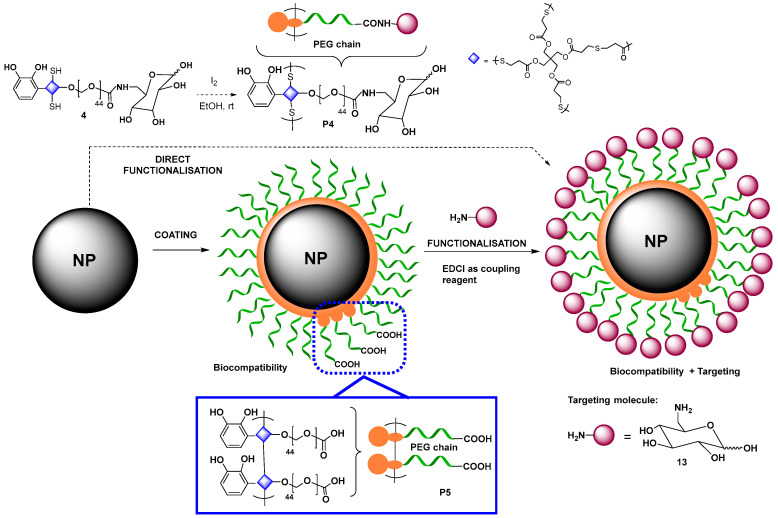
Strategies followed to obtain the functionalized coated Fe_3_O_4_@**P5**–glucose NPs with biocompatibility and possible targeting properties.

To validate both our coating capabilities on different nanoparticle families and their subsequent use for additional biofunctionalization, we tackled the synthesis of Fe_3_O_4_ NPs functionalized with **P4**; these are sugars recognized in Biology because of their barrier-crossing properties (Figure 5). Sadly, all the attempts to synthesize the precursor compound **4** failed (see Scheme S1 in the Supplementary Material). In the last synthetic stages, after incorporating the catechol and the protected sugar moieties, the resulting product proved to be unstable. Alternatively, we planned the first coating of the NPs with the oligomeric material **P5,** derived from cat-PEG-COOH bis thiol **5**, to later couple the carboxylic acid residues with the suitable aminosugar derivative (Figure 5). Next, the first magnetic NPs stabilized with oleic acid (Fe_3_O_4_@OA NPs) were obtained through the thermal decomposition of Fe(acac)_3_ in the presence of oleic acid in a mixture of solvents. This procedure ensured a precise nanoparticle size (average 8 nm in diameter) and monodispersity, as confirmed by the electron microscopy (TEM) images [48]. Next, the Fe_3_O_4_@OA NPs were suspended in CHCl_3_ and coated with **P5**, previously dissolved in the same solvent. Despite many attempts, isolating the coated NPs through centrifugation proved unfeasible.

However, upon mixing the suspension with water, the NPs were transferred to the aqueous phase, unlike the uncoated NPs, which remained in the organic phase. This already represents a qualitative demonstration of the nanoparticle coating. The last step was the functionalization, performed by covalently attaching the carboxylic acid residues of the Fe_3_O_4_@**P5** NPs with a suitable aminosugar derivative. Before this step, the reaction was optimized with a simple allylamine model, whose incorporation could be easily monitored by ^1^H NMR. Hence, a freshly prepared suspension of Fe_3_O_4_@**P5** NPs in CHCl_3_ was treated with the coupling reagent EDCI and allylamine overnight. The resulting Fe_3_O_4_@**P5**-allylamide NPs were transferred to an aqueous phase and evaporated to dryness. The resulting solid was dispersed in deuterated methanol with some drops of DCl and left to stand overnight in this acidic medium to degrade the NPs. Finally, a ^1^H-NMR spectrum was recorded, revealing the presence of all the expected allyl signals coming from the linked allylamine, albeit slightly shifted to a lower field, according to the formation of the amide bond (see Supplementary Material, S7).

Finally, this synthetic procedure was successfully repeated with the protected aminofuranose derivative **12**, obtaining the Fe_3_O_4_@**P5**-ProtecFuranoseAmide NPs (as confirmed by the ^1^H NMR spectrum of the degraded NPs, see Supplementary Material, S7). However, the subsequent treatment of Fe_3_O_4_@**P5**-ProtecfuranoseAmide NPs in water with different acidic conditions to transform the attached sugar derivative fragments into the corresponding glucose units did not work. Under mildly acidic conditions, the protecting groups were not fully removed, while in stronger acidic media, an NP degradation was found. Therefore, the coupling was repeated using free amino-glucopyranose **13** (derived from the protected amino-glucofuranose **12**, see Supplementary Material). In this way, the desired Fe_3_O_4_@**P5**-GlucoseAmide NPs (Figure 1) were directly obtained, as confirmed by the ^1^H- and ^13^C NMR spectra (see Supplementary Material, S7). The DLS and zeta ζ-potential measurements at pH 7,4 (PBS buffer) showed aggregates around 360 nm and α ζ-potential of −13.9 mv, as well as 930 nm with a ζ-potential value of 3.3 mv for the Fe_3_O_4_@**P5** NPs and Fe_3_O_4_@**P5**-Glucose NPs, respectively. These substantial ζ-potential changes and the tendency to form larger aggregates for the Fe_3_O_4_@**P5**-Glucose NPs agree with the blocking of the carboxylic acid residues with the glucose units. Interestingly, the addition of the bovine serum albumin (BSA 10%) in the same PBS buffer to simulate physiological environments stabilized the monodispersed Fe_3_O_4_@**P5**-Glucose NPs on smaller aggregates around 100 nm, even though their ζ-potential was close to 0 mv.

### 3.4. Multifunctional Coatings

**Figure 6:**
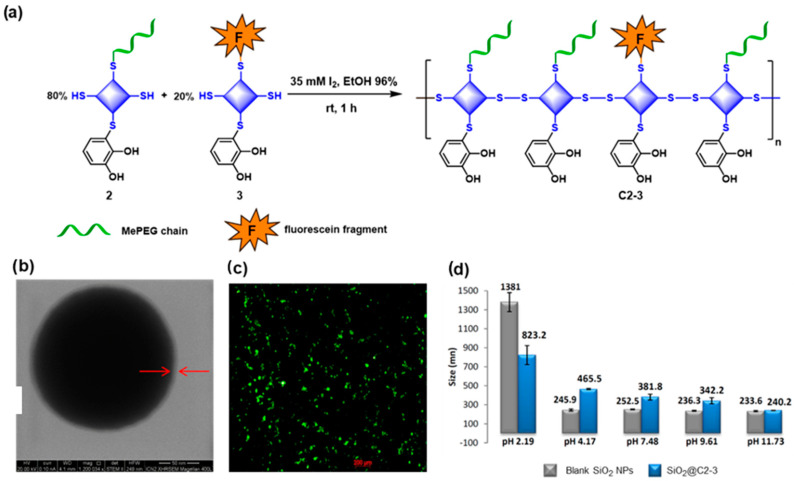
(**a**) Copolymerization of synthesized building blocks **2** and **3** to obtain a copolymer, **C2**–**3**, which is biocompatible, hydrophilic, and fluorescent, and is suitable for biomedical applications; (**b**) STEM image of coated MSNPs@**C2**–**3** showing a lighter outer layer. (**c**) Image taken from an inverted optical/fluorescence microscope in fluorescence mode with an Alexa Fluor 488 filter of MSNPs@**C2**–**3** showing fluorescence. (**d**) Representation of NPs’ sizes at different pH taken from DLS analyses.

Finally, a multifunctional coating (referred to, from now on, as **C2**–**3**) was obtained with a 48% yield by copolymerization of the cat-PEG **2** (80%) and the fluorescein-functionalized unit **3** (20%), using the polymerization protocol previously described (Figure 6a). The incorporation of both functionalities was confirmed by ^1^H NMR, with aromatic signals from the fluorescein in the 6.5–8.0 ppm range and the characteristic PEG signal at ~3.6 ppm (see Supplementary Materials for full details). The posterior MSNP coating was confirmed by STEM analysis, which revealed a thin lighter layer around the surfaces (Figure 6b), and the fluorescence emission was detected by an optical fluorescent microscope equipped with an Alexa Fluor 488 filter (Figure 6c). The DLS measurements confirmed a colloidal stability increase at higher pHs, resulting in smaller sizes due to improved electrostatic stabilization (Figure 6d). The MSNP@**C2**–**3** formed larger aggregates than the non-treated MSNPs between pH 4 and pH 10.

**Figure 7:**
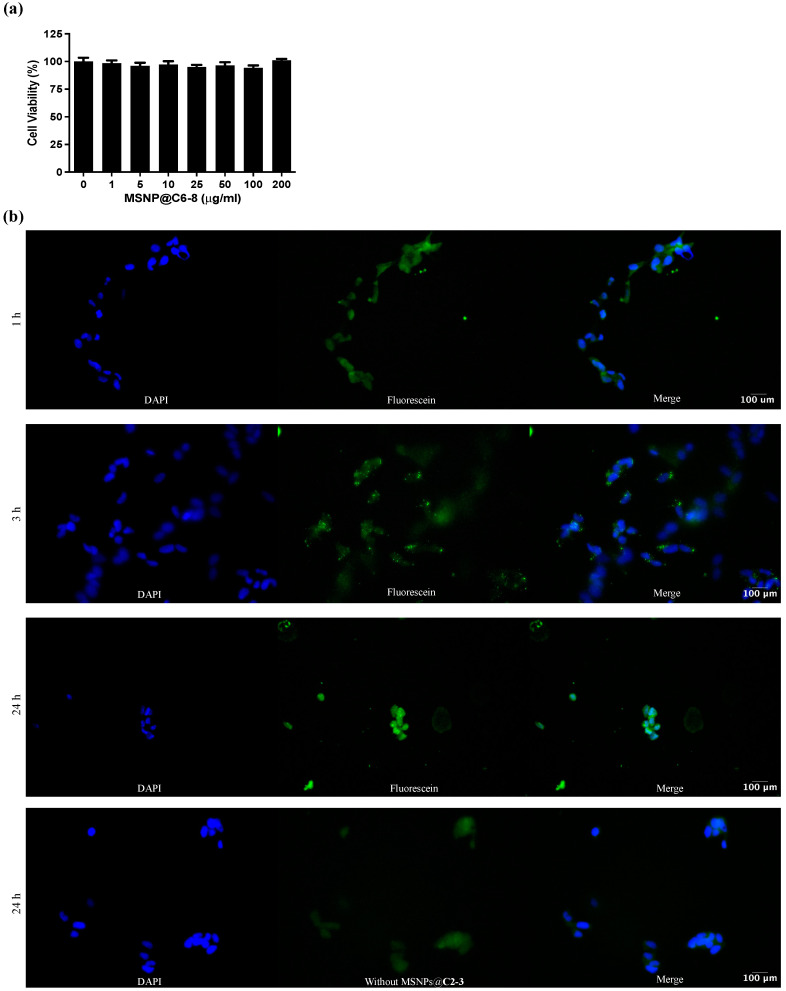
(**a**) Effect of MSNPs@**C2**–**3** on the cell viability of SH-SY5Y cells (3000 cells per well). These cells were incubated for 24 h in the presence of indicated concentrations of MSNPs@**C2**–**3**. Cell viability is expressed as a percentage compared to an untreated control. Values are presented as mean ± standard error of the mean (n = 3). (**b**) Internalization assay on SH-SY5Y cells at 1 h, 3 h, 24 h, and 24 h without MSNPs@**C2**–**3**. Blue color shows 4′,6-diamidino-2-phenylindole (DAPI) staining of cell nuclei. The green color shows fluorescein staining of MSNPs@**C2**–**3**. Scale: 100 µm.

Finally, an internalization assay was conducted on SH-SY5Y cells. This involved adding MSNPs@**C2**–**3** (200 µg/mL) to six-well plates containing 10^5^ cells per well. At different time points (1, 3, 6, 24 h), the cells were fixed and stained (Figure 7b).

After 1 h, MSNPs@**C2**–**3** were observed within the cells. At 3 h, these nanoparticles were not only internalized but also exhibited an organized distribution within the cellular compartments. By 24 h, nanoparticles were additionally detected in the nuclei of the cells, showing an organized arrangement. In contrast, cells without nanoparticles showed no fluorescence. Furthermore, the cytotoxic effect of the MSNPs@**C2**–**3** on the SH-SY5Y cells was examined by treating them with varying concentrations of MSNPs@**C2**–**3** for 24 h. These coated NPs did not exhibit a cytotoxic effect compared to the control at any of the concentrations tested (Figure 7a).

## 4. Conclusions

We have developed a novel mussel-inspired strategy to obtain multifunctional PEG-based biocoatings by deploying the polymerization of catechol-based functionalized units using disulfide bridges under mild oxidative conditions. Thanks to its universal character, we successfully coated two families of NPs, mesoporous silica NPs (MSNPs) and magnetic NPs (MNPs, Fe_3_O_4_ NPs), both of which are receiving increasing recognition for their biomedical uses. The PEG coating confers biocompatibility and colloidal stability to the NPS and is used as a primer for further functionalization through the carboxylic acid moieties with bioactive groups. In this case, and as a representative example, we introduced glucose units with potential barrier-crossing capabilities. Finally, and as a proof of concept, we have also shown the possibility of achieving multifunctional coatings combining PEG chains and fluorescein fragments, allowing for the optical tracking of additional cell internalization, with excellent biocompatibility.

## Data Availability

Data are contained within the article.

## Supplementary Materials

The following supporting information can be downloaded at: https://www.mdpi.com/article/10.3390/biomimetics9090531/s1: S1: experimental part of synthesis of monomers; S2: experimental part synthesis of amino glucopyranose; S3: experimental part of synthesis of intermediate **21**; S4: ^1^H, ^13^C NMR and IR spectra of new compounds; S5: ^1^H NMR and IR spectra of oligomers and copolimers; S6: GPC spectra; S7: other ^1^H, ^13^C NMR spectra.
