# Supplementary material for: Mussel-Inspired Multifunctional Polyethylene Glycol Nanoparticle Interfaces
Source: Biomimetics (Basel). 2024 Sep 4;9(9):531. doi: 10.3390/biomimetics9090531 (PMC11429798; doi:10.3390/biomimetics9090531)
Supplement: Supplementary file 1 [file biomimetics-09-00531-s001.zip › biomimetics-3072959-supplementary.pdf]

Supporting Information for Mussel-Inspired Multifunctional Polyethylene Glycol Nanoparticle Interfaces

Carolina Casagualda [1,2], Alba López-Moral [1,2], Paula Alfonso-Triguero [3], Julia Lorenzo [3,4], Ramon Alibés [2], Félix Busqué [2,*] and Daniel Ruiz-Molina [1,*]

[1] Catalan Institute of Nanoscience and Nanotechnology (ICN2), CSIC, and The Barcelona Institute of Science and Technology (BIST), Campus UAB, Bellaterra, 08193 Barcelona, Spain
[2] Departament de Química, Universitat Autònoma de Barcelona, Bellaterra, 08193 Barcelona, Spain
[3] Institut de Biotecnologia i de Biomedicina and Departament de Bioquímica i Biologia Molecular, Universitat Autónoma de Barcelona, Bellaterra, 08193 Barcelona, Spain
[4] Centro de Investigación Biomédica en Red (CIBER), Bioingeniería, Biomateriales y Nanomedicina, 08193 Cerdanyola del Vallès, Spain
* Correspondence: felix.busque@uab.cat (F.B.); dani.ruiz@icn2.cat (D.R.-M.)

Table of contents

	Page
S1. Synthesis of monomers **1**, **2** and **5** (Experimental part)	S2
S2. Synthesis of amino-glucopyranose **13** (Experimental part)	S3
S3. Synthesis of intermediate **20**, precursor of monomer **4** (Experimental part)	S4
S4. ^{1}H, ^{13}C NMR and IR spectra of new compounds	S9
^{1}H, ^{13}C NMR and IR spectra of compound **19**	S9
^{1}H, ^{13}C NMR and IR spectra of compound **9**	S11
^{1}H, ^{13}C NMR and IR spectra of compound **10**	S13
^{1}H, ^{13}C NMR and IR spectra of compound **1**	S15
^{1}H, ^{13}C NMR and IR spectra of compound **2**	S17
^{1}H, ^{13}C NMR and IR spectra of compound **5**	S19
^{1}H, ^{13}C NMR and IR spectra of compound **15**	S21
^{1}H, ^{13}C NMR and IR spectra of compound **16**	S23
^{1}H, ^{13}C NMR and IR spectra of compound **12**	S25
^{1}H and ^{13}C NMR spectra of compound **17**	S27
^{1}H NMR spectrum of compound **20**	S28
^{1}H and ^{13}C NMR and IR spectra of compound **13**	S29
S5 1H NMR and IR spectra of oligomers and copolimers	S31
^{1}H NMR and IR spectra of oligomer **P1**	S31
^{1}H NMR and IR spectra of oligomer **P2**	S32
^{1}H NMR and IR spectra of oligomer **P5**	S33
^{1}H NMR and IR spectra of copolimer **C2-C3**	S34
S6 GPC spectra	S35
S7 ^{1}H NMR spectra organic part of degraded Fe_3O_4@**P5-Amides** NPs	S36
^{1}H NMR spectra of the organic part of degraded Fe_3O_4@**P5-Allylamide** NPs	S36

^{1}H NMR spectra of the organic part of degraded Fe_3O_4@**P5-ProtectedSugar amide** NPs	S37
^{13}C NMR spectra of the organic part of degraded Fe_3O_4@**P5-Sugar amide** NPs	S38

S1. Synthesis of monomers **1**, **2** and **5**

Monomer 1. To a solution of the *S*-catechol tris-thiol **7**[1] (73 mg, 0.11 mmol) in dry toluene (1 mL) with DMPP (11 µL, 0.01 mmol), heterobifunctional PEG derivative **8** (102 mg, 0.05 mmol) was added, and the mixture stirred at 30 °C for 4 h. The solvent was removed under vacuum, the crude redissolved in the minimum volume of CH_2Cl_2 and Et_2O was added until the solution became turbid. The solution was stored in the freezer overnight and the white precipitate was filtered, washed with cold Et_2O and dried under high vacuum to furnish the final catechol-PEG derivative **1** as a white powder (103 mg, 0.04 mmol, 78% yield). **^{1}H NMR** (360 MHz, $CDCl_3$) δ 6.97-6.88 (m, 2H, H-7, H-9), 6.75 (t, *J* = 7.9 Hz, 1H, H-8), 4.19 (m, 10H, H-2, H-2'), 4.09 (s, 2H, H-12), 3.74-3.50 (bs, 180H, -$(CH_2CH_2O)_n$-), 3.08-2.51 (m, 20H, H-4, H-5, H-4', H-5'), 1.63 (t, *J* = 8.3 Hz, 2H, -SH).**^{13}C NMR** (90.5 MHz, $CDCl_3$) δ 171.2 (C-3, C-13), 151.9 (C-10), 139.5 (C-11), 130.3 (C-7), 126.0 (C-6), 120.8 (C-8), 117.0 (C-9), 70.8 (-$(CH_2CH_2O)_n$-), 62.1 (C-2), 38.2 (C-1), 31.0 (C-4, C-4'), 19.6 (C-5, C-5').

Monomer 2. To a solution of allyl-functionalised MePEG-allyl **10** (102 mg, 0.13 mmol) in dry toluene (6 mL), AIBN (8 mg, 0.05 mmol) was added under N_2 atmosphere. The mixture was heated at reflux temperature and a solution of *S*-catechol tris-thiol **7** (96 mg, 0.16 mmol) in dry toluene (9 mL) was added dropwise. The reaction mixture was heated at reflux temperature for 24 h. After cooling at rt, the solvent was removed under reduced pressure to afford an oil which was dissolved in the minimum quantity of CH_2Cl_2 and Et_2O was added until cloudiness appeared. The solution was stored in the fridge overnight and the resulting precipitate filtered, washed five times with cold Et_2O and dried at high vacuum to give product **2** as a white powder (209 mg, 0.08 mmol, 62% yield). **^{1}H NMR** (360 MHz, $CDCl_3$) δ 7.13-6.68 (m, 3H, H-8, H-9, H-10), 4.17 (s, 8H, H-2), 3.70-3.52 (bs, 200H, -$(CH_2CH_2O)_n$-), 3.37 (s, 3H, H-13), 3.11-2.50 (m, 18H, H-4, H-5, H-4', H-5'), 1.61 (t, *J* = 8.2 Hz, 2H, -SH), 1.31 (q, *J* = 7.2 Hz, 2H, H-6). **^{13}C NMR** (125.8 MHz, $CDCl_3$) δ 171.1 (C-3), 162.5 (C-11), 144.6 (C-12), 122.3 (C9), 119.7 (C-8), 114.1 (C-7), 109.2 (C-10), 70.4 (-$(CH_2CH_2O)_n$-), 61.6 (C-2), 59.1 (C-13), 41.8 (C-1), 38.3 (C-4'), 36.5 (C-4), 31.5 (C-5), 27.9 (C-6), 19.6 (C-5').

Monomer 5. To a solution of the *S*-catechol tris-thiol **7** (343 mg, 0.57 mmol) in dry toluene (14 mL) with DMPP (61 µL, 0.04 mmol), heterobifunctional PEG derivative **9** (204 mg, 0.10 mmol) was added, and the mixture stirred at 30 °C for 5 h. The solvent was removed under vacuum, the crude redissolved in the minimum volume of CH_2Cl_2 and Et_2O was added until the solution

became turbid. The solution was stored in the freezer overnight and the white precipitate was filtered, washed with cold Et_2O and dried under high vacuum to furnish the final cat-PEG derivative **5** as a white powder (176 mg, 0.07 mmol, 67% yield). **^{1}H NMR** (360 MHz, $CDCl_3$) δ 6.97-6.88 (m, 2H, H-7, H-9), 6.75 (t, *J* = 7.9 Hz, 1H, H-8), 4.19 (m, 10H, H-2, H-2'), 4.09 (s, 2H, H-12), 3.74-3.50 (bs, 180H, -$(CH_2CH_2O)_n$-), 3.08-2.51 (m, 20H, H-4, H-5, H-4', H-5'), 1.63 (t, *J* = 8.3 Hz, 2H, -SH). **^{13}C NMR** (90.5 MHz, $CDCl_3$) δ 171.2 (C-3, C-13), 151.9 (C-10), 139.5 (C-11), 130.3 (C-7), 126.0 (C-6), 120.8 (C-8), 117.0 (C-9), 70.8 (-$(CH_2CH_2O)_n$-), 62.1 (C-2), 38.2 (C-1), 31.0 (C-4, C-4'), 19.6 (C-5, C-5').

S2. Synthesis of amino-glucopyranose **13**

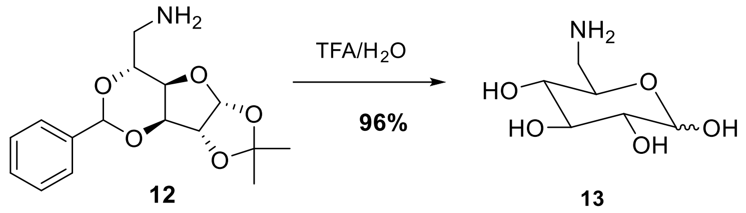

To a stirred solution of protected amino-glucofuranose derivative **12** (28.3 mg, 0.09 mmol) in MilliQ H_2O (6 mL), TFA (3 mL, 39.96 mmol) was added. The mixture was stirred at rt for 1.5 h. The reaction mixture was washed with CH_2Cl_2 (3 x 10 mL) and concentrated under vacuum. The deprotected amino-glucopyranose derivative **13** was obtained as a colourless oil (15.1 mg, 0.09 mmol, 96% yield). **^{1}H NMR** (360 MHz, CD_3OD) δ 5.11 (d, *J* = 3.6 Hz, 1H, H-1), 4.48 (d, *J* = 7.8 Hz, 1H, H-5), 3.93 (td, *J* = 9.2, 3.2 Hz, 1H, H-2), 3.63 (t, *J* = 9.2 Hz, 1H, H-6), 3.45 (td, *J* = 9.2, 3.1 Hz, 1H, H-3), 3.36 (d, *J* = 3.8 Hz, 1H, H-6'), 3.33 (m, 1H, H-4), 3.14 (m, 3H, H-8, H-9, H-10), 2.96 (m, 1H, H-7). **^{13}C NMR** (90.5 MHz, CD_3OD) δ 98.4 (C-1), 77.6 (C-3), 76.0 (C-2), 74.4 (C-5), 73.5 (C-4), 42.2 (C-6). **HRMS** (HR-EI) Calcd. for $[C_6H_{14}NO_5]^+$: 180.0872; found: 180.0869.

S3. Towards the synthesis of compound **20** immediate precursor of cat-PEG-sugar **4**

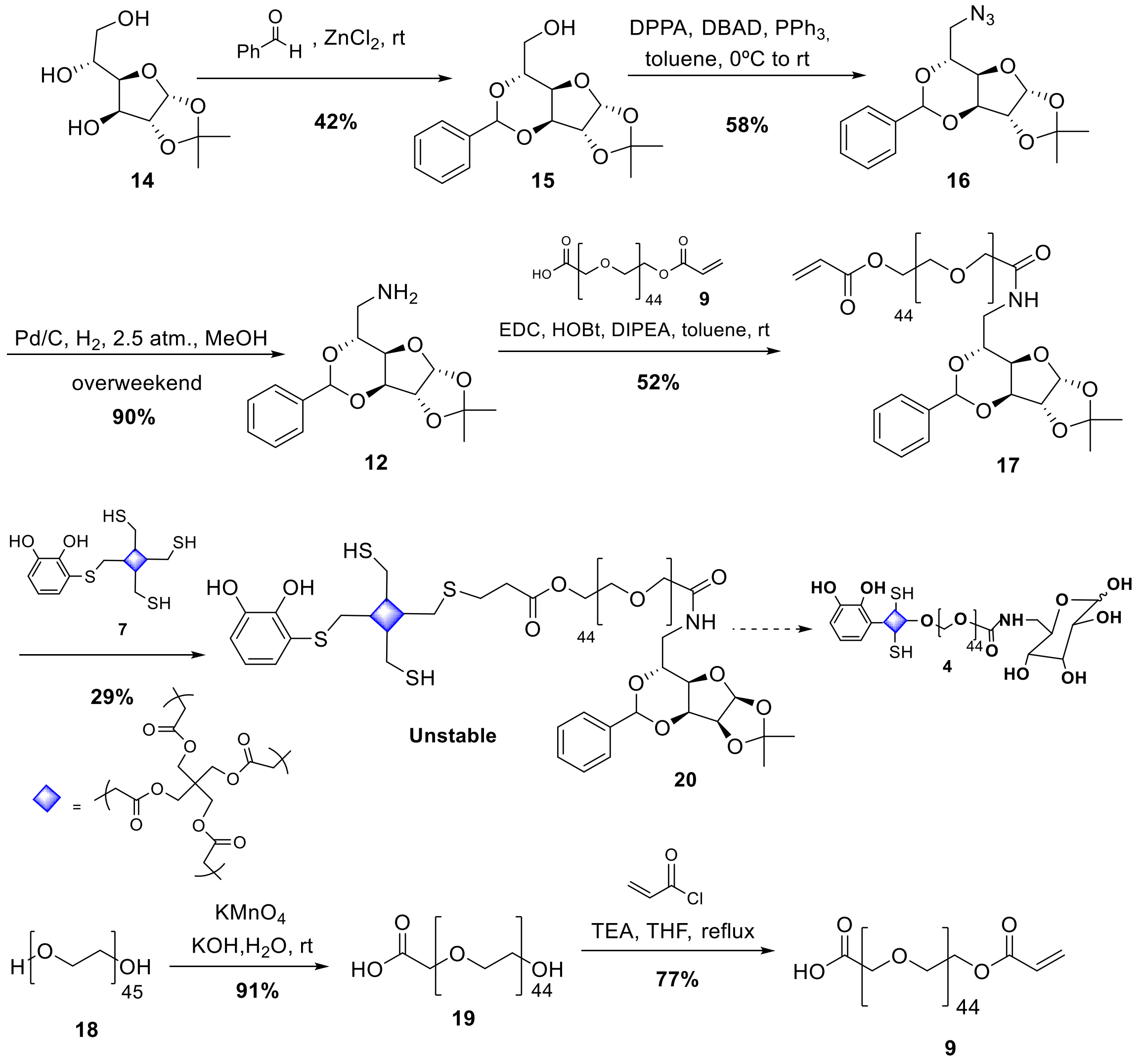

Scheme S1. Synthesis of compound **20**, immediate precursor of cat-PEG-sugar **4**.

The synthesis of compound **4** was approached as outlined in Scheme S1. Thus, the preparation started from commercial 1,2-*O*-isopropylidene-α–β-glucofuranose **14**, protecting the remaining diol as the corresponding benzaldehyde ketal according to literature to render the alcohol **15** in 42% yield. This alcohol **15** was subsequently submitted to Mitsunobu conditions using the azide anion equivalent diphenyl phosphorazidate (DPPA), affording azide **16** in a 58% yield, which was hydrogenated under standard conditions to yield the corresponding amine **12** in a 90% yield. Then, this amine **12** was coupled with the acid PEG derivative **9** using EDC as coupling agent in the presence of HOBt and DIPEA, to render the corresponding amide intermediate **17** in 52% yield. The acid PEG intermediate **9** was obtained from polyethylene glycol **18** (2000 average molecular mass) by oxidation with $KMnO_4$ of one of the free hydroxyls to the corresponding carboxylic acid to afford hydroxy-acid **19** in a 91% yield, followed by esterification of the remaining hydroxyl with acryloyl chloride in the presence of TEA, to afford **9** in 77% yield. Finally,

intermediate **17** was allowed to react through a thia-Michael reaction with the catechol tris-thiol **7**, already described,[1] using DMPP as catalyst to afford **20** in29% yield. This compound resulted unstable, even at low temperature and argon atmosphere, and impossible to purify or use it in further reactions.

((3a*S*,3b*R*,7*S*,7a*S*,8a*S*) 2,2-dimethyl-5-phenyltetrahydro-7 *H*-[1,3] dioxolo [4',5':4,5] furo [3,2-d][1,3]dioxin-7-yl)methanol, 15.[2] 1,2-*O*-isopropylidene-α-D-glucofuranoside (2.200 g, 9.99 mmol) and zinc chloride (2.860 g, 20.99 mmol, 2,1 eq.) were mixed in benzaldehyde (8.5 mL, 83.4 mmol, 8,3 eq.) at rt. The mixture was stirred for 5 h with 1.4 g of molecular sieves (4 Å) and then diluted with ethyl acetate (7mL). The resulting solution was sequentially washed with $NaHCO_{3(sat.)}$ and brine (30 mL x 3). Then, the organic layers were dried over anhydrous sodium sulphate, filtered, evaporated under vacuum, and finally recrystallized from hexane at 4 °C to afford the previously reported protected glucofuranose **15** in 42% yield. **^{1}H NMR** (360 MHz, MeOD) δ 1.33 (s, 3H, H-2), 1.48 (s, 3H, H-1), 3.95 (d, *J*=1.24 Hz, 2H, H-8b, H-5), 4.16 (d, *J*=1.16, 2H, H-8a, H-7), 4.54 (d, *J*=2.24 Hz, 1H, H-6), 4.64 (d, $J_{4\text{-}3}$=3.75 Hz, 1H, H-4), 5.92 (s, 1H, H-9), 5.99 (d, $J_{3\text{-}4}$=3.72 Hz, 1H, H-3), 7.33 (m, 3H, H-11, H-12, H-13), 7.46 (m, 2H, H-10, H-14). **Mp** 148-151 °C (from hexane).

((3a*S*,3b*R*,7*S*,7a*S*,8a*S*)-7-(azidomethyl)-2,2-dimethyl-5- phenyltetrahydro-7*H*-[1,3] dioxolo [4',5':4,5]furo[3,2-d][1,3]dioxine, 16. DBAD (0.624 g, 2.71 mmol, 1.4 equivalents) was added into a solution of PPh_3 (0.710 g, 2.71 mmol, 1.4 equivalents) in 11 mL of dry CH_2Cl_2 at 0 °C and stirred for 1 h under argon atmosphere. Afterwards, DPPA (0.430mL, 1.94 mmol, 1 equivalents) and a solution of sugar derivative **15** (0.597g, 1.94 mmol, 1 eq.) in 9 mL of dry CH_2Cl_2 were added to the previous solution under anhydrous conditions. The resulting solution was left at rt overnight. The crude was filtered over Celite® and the solvent removed under vacuum. The crude material was purified by column chromatography through silica gel with hexane:EtOAc (10:1) to afford the product **16** as a white solid in 58% yield. **^{1}H NMR** (360 MHz, MeOD) δ 1.33 (s, 3H, H-2), 1.48 (s, 3H, H-1), 3.52 (dd, J_1=13.35 Hz, $J_{8b\text{-}7}$= 4.93 Hz, 1H, H-8b), 4.02 (m, J_1=13.46 Hz, $J_{5\text{-}6}$= 2.27 Hz, $J_{8a\text{-}7}$= 9.12 Hz, 2H, H-8a, H-5), 4.34 (dd, $J_{7\text{-}8a}$=9.08 Hz, $J_{7\text{-}8b}$= 4.86 Hz, 1H, H-7), 4.54 (d, $J_{6\text{-}5}$=2.32 Hz, 1H, H-6), 4.66 (d, $J_{4\text{-}3}$= 3.73 Hz, 1H, H-4), 5.88 (s, 1H, H-9), 5.99 (d, $J_{3\text{-}4}$= 3.69 Hz, 1H, H-3), 7.35 (m, 3H, H-11, H-12, H-13) 7.46 (m, 2H, H-10, H-14). **^{13}C NMR** (360 MHz, MeOD): δ 26.3 ppm (C-2), 26.9 (C-1), 50.6 (C-8), 74.2 (C-5), 74.4 (C-7), 78.5 (C-6), 85.1 (C-4), 94.6 (C-9), 106. (C-3), 113.1 (C-14), 127.4 (2C, C-10), 129.1 (2C, C-11), 130.0 (C-12), 139.1 (C-13). **Mp** 118-120 °C (from hexane-EtOAc). **HRMS** (HR-EI) Calcd. for $[C_{16}H_{19}N_3O_5Na]^+$: 356.1217; found: 356.1222.

((3a*S*,3b*R*,7*S*,7a*S*,8a*S*) -2,2-dimethyl-5-phenyltetrahydro-7 *H*-[1,3]dioxolo [4',5':4,5]furo [3,2-d][1,3]dioxin-7-yl)methanamine, 12. Intermediate **16** (0.426 g, 1.3 mmol) was dissolved in MeOH (90 mL). Pd/C (43 mg) was added, and the resulting mixture was stirred for 15 minutes. The hydrogenation system was connected in 2.5 atm of pressure for 3 days. The solution was filtered over Celite® and the filtrate was evaporated under vacuum to afford the resulting compound **17** in 90% yield. ^{1}**H NMR** (360 MHz, MeOD) δ 1.32 (s, 3H, H-2), 1.48 (s, 3H, H-1), 2.87 (dd, $J_{8b\text{-}8a}$=12.6 Hz, $J_{8b\text{-}7}$= 5.32 Hz, 1H, H-8b), 3.24 (m, 1H, H-8a), 4.04 (s, 1H, H-5), 4.18 (dd, $J_{7\text{-}8a}$=9.9 Hz, $J_{7\text{-}8b}$= 5.30 Hz, 1H, H-7), 4.49 (s, 1H, H-6), 4.65 (d, $J_{4\text{-}3}$= 3.71 Hz, 1H, H-4), 5.80 (s, 1H. H-9), 5.99 (d, $J_{3\text{-}4}$= 3.63 Hz, 1H, H-3), 7.35 (m, 3H, H-11, H-12, H-13), 7.49 (m, 2H, H-10, H-14). ^{13}**C NMR** (360 MHz, MeOD): δ 26.3 ppm (C-2), 26.9 (C-1), 39.4 (C-8), 73.4 (C-5), 74.04 (C-7), 78.36 (C-6), 85.1 (C-4), 94.3 (C-9), 106.5 (C-3), 113.2 (C-14), 127. (2C, C-10), 129.1 (2C, C-11), 130.1 (C-12), 139.1 (C-13). **HRMS** (HR-EI) Calcd. for $[C_{16}H_{22}NO_5]^+$: 308.1496; found: 308.1496.

2-polyethylenglicol acetic acid 19. Commercial PEG-2000 (2.503 g, 1.25 mmol) and KOH (1.307g, 19.75 mmol, 15.8 eq.) were dissolved in 37.5 mL of water. $KMnO_4$ (0.387 g, 2.375 mmol, 1.4 eq.) was added and the reaction mixture was stirred overnight at rt. The resulting brown suspension of MnO_2 was filtered over Celite and HCl (37%) was added to the filtrate until acidic pH (pH ≈ 4). The product was extracted into CH_2Cl_2. The organic layers were dried with anhydrous sodium sulphate, filtered, and evaporated under vacuum to reduce the volume. Diethyl ether was added to the resulting oil until the solution became turbid. The solution was stored in the freezer overnight and the white precipitate was filtered, washed with cold diethyl ether, and dried under vacuum to obtain the final product in 91% yield. ^{1}**H NMR** (360 MHz, $CDCl_3$) δ 3.63 (s, 178 H, -CH_2-CH_2-), 4.13 (s, 2H, H-1).

2-(ω-acryloyl-polyethylenglicol) acetic acid 9. 2-polyethylenglicol acetic acid **19** (0,830 g, 0,409 mmol) in anhydrous THF (42 mL) was stirred for 30 min with 0.800 g of molecular sieves under Ar atmosphere at rt. Acryloyl chloride (134 µL, 1.64 mmol, 4 equiv) and anhydrous TEA (0.28 mL, 2.05 mmol, 5 equiv) were added in this order. The reaction mixture was stirred for 4 h at the reflux temperature. The reflux was removed, and the crude was left to rt. Next, the suspension was filtered over celite to remove the white solid and THF was evaporated. The resulting oil was dissolved in CH_2Cl_2 (20 mL) and acidified with acetic acid until acidic pH. The solution was washed with 20 mL of brine six times. Then, the organic layers were dried with anhydrous sodium sulphate and filtered. The solvent was removed under vacuum and the resulting oil dissolved in THF (20 mL). Afterward, cold hexane was added until the solution became turbid and stored in the freezer for 1 h. Finally, compound **19** was recovered by filtration (77% yield). ^{1}**H NMR** (360 MHz, $CDCl_3$) δ 3.63 (s, 178 H, -CH_2-CH_2-), 4.13 (s, 2H, H-1), 4.31 (t, *J*= 3.60, 2H, H-2), 5.82 (dd, $J_{5a\text{-}5b}$= 0.97, $J_{5a\text{-}4}$= 10.5, 1H, H-5), 6.15 (dd, $J_{4\text{-}5a}$= 10.5, $J_{4\text{-}5b}$= 17.3, 1H. H-4), 6.43 (dd, $J_{5b\text{-}5a}$= 1.3, $J_{5b\text{-}4}$=

17.4, 1H, H-5b). **^{13}C NMR** (100.6 MHz, $CDCl_3$) δ 172.3 (C-6), 166.5 (C-3), 131.3 (C-1), 128.6 (C-2), 70.8 (-$(CH_2CH_2O)_n$-), 69.4 (C-5), 63.9 (C-4). **Mp** 48-50 °C (from THF-hexane).

α-2-(2-((((3a*S*,3b*R*,7*S*,7a*S*,8a*S*)-2,2-dimethyl-5-phenyltetrahydro-7H-[1,3]dioxolo[4',5':4,5]-furo[3,2-d][1,3]dioxin-7-yl)methyl)amino)- ω- acryloyl- poly(ethylene glycol) 2000, 17. A solution of 2-(ω-acryloyl-polyethylenglicol) acetic acid **9** (0.205 g, 0.098 mmol) in 5 mL of dry DCM was stirred with 0.3 g of molecular sieves. After 30 minutes, EDC 98% (53 µL, 0.294 mmol, 3 eq.), DIPEA (74 µL, 0.441 mmol, 4.5 eq.) and HOBt (0.045 g, 0.294 mmol, 3 equiv) were added. Finally, a solution of protected aminofuranose derivative **16** (0.060 g, 0.196 mmol, 2 equiv) in 5 mL of dry CH_2Cl_2 was added to the first one and stirred overnight under argon atmosphere. The crude was filtered and washed four times with brine. The organic phase was dried with anhydrous Na_2SO_4, filtered, and evaporated under vacuum. The resulting oil was dissolved in a 1:1 mixture of CH_2Cl_2 and EtOAc. Diethyl ether was added until the solution became turbid. The solution was stored overnight in the freezer and the white precipitate was filtered, washed with cold diethyl ether, and dried under high vacuum to afford **17** in 52% yield. **^{1}H NMR** (360 MHz, MeOD) δ 1.32 (s, 3H, H-2), 1.48 (s, 3H, H-1), δ 3.63 (s, 177 H, -CH_2-CH_2-, H-8b and H-16), 4.00 (m, 2H, H-8a and H-15), 4.05 (s, 1H, H-5), 4.29 (t, 2H, H-17), 4.35 (dd, J_1=9.4 Hz, J_2= 6.59 Hz, 1H, H-7), 4.56 (s, 1H, H-6), 4.66 (d, $J_{4\text{-}3}$= 3.46,1H, H-4), 5.86 (s, 1H, H-9), 5.88 (dd, $J_{19a\text{-}19b}$= 0.97, $J_{19a\text{-}18}$= 10.5, 1H, H-19a), 6.01 (d, J= 3.63 Hz, 1H, H-3), , 6.18 (dd, $J_{18\text{-}19a}$= 10.5, $J_{18\text{-}19b}$= 17.3, 1H. H-18), 6.43 (dd, $J_{19b\text{-}19a}$= 1.3, $J_{19b\text{-}18}$= 17.4, 1H, H-19b), 7.35 (m, 3H, H-11, H-12, H-13), 7.49 (m, 2H, H-10, H-14).**^{13}C NMR** (360 MHz, MeOD) δ 25.1 (C-2), 25.7 (C-1), 37.3 (C-8), 63.5 (C-16), 68.7 (C-15), 70.2 (89C, C-21 i (-CH_2CH_2O-$)_{n\text{-}2}$), 71.6 (C-5), 72.7 (C-7), 76.9 (C-6), 83.8 (C-4), 92.9 (C-9), 105.1 (C-3), 111.6 (C-14), 126.1 (C-10), 127.7 (C-11), 128.1 (C-18), 128.5 (C-12), 130.4 (C-19), 138.1 (C-13), 166.1 (C-17), 171.7 (C-20). **Mp** 40-42 °C (from diethyl ether).

2-(((3-((2,3-dihydroxyphenyl)thio)propanoyl)oxy)methyl)-2-(1-((3a*R*,3b*S*,7a*S*,8a*R*)-2,2-dimethyl-5-phenyltetrahydro-7H-[1,3]dioxolo[4',5':4,5]furo[3,2-d][1,3]dioxin-7-yl)-3,9,15-trioxo-5,8,16-trioxa-12-thia-2-azaheptadecan-17-yl)propane-1,3-diyl bis(3-mercapto-propanoate), 20. To a solution of catechol tris-thiol **7**, already described,[1] (615 mg, 1 mmol) in dry toluene and under inert atmosphere was added dimethylphenylphosphine DMPP 97% (5.2 µL, 0.036 mmol). To this solution, intermediate **17** (318 mg, 0.16 mmol) was added, and the mixture quickly agitated at 30 °C for 6.5 h. The solvent was removed under vacuum and the crude re-dissolved in the minimal amount of CH_2Cl_2. Diethyl ether was added until the solution became turbid. The solution was stored in the freezer overnight and the white precipitate was filtered, washed with cold ether, and dried under high vacuum to obtain a wax in a 29% yield, which resulted unstable and rapidly degraded. **^{1}H NMR** (360 MHz, $CDCl_3$) δ 1.32-1.60 (m, 8H, H-2, H-1, H-19), 2.48-3.13 (m, 20H, H-16), 3.63 (s, 177 H, -CH_2-CH_2O-), 4.02 (m, 3H, H-8 and H-5), 4.12 (s,

10H, H-15, H-17), 4.35 (m, 1H, H-7), 4.56 (s, 1H, H-6), 4.66 (d, $J_{4\text{-}3}$= 3.46,1H, H-4), 5.86 (s, 1H, H-9), 6.01 (d, J= 3.63 Hz, 1H, H-3), 6.77-7.02 (m, 3H, H-18), 7.35 (m, 3H, H-11, H-12, H-13), 7.49 (m, 2H, H-10, H-14).

1. Casagualda, C.; Mancebo-Aracil, J.; Moreno-Villaécija, M.A.; López-Moral, A.; Alibés, R.; Busqué, F.; Ruiz-Molina, D. Mussel-Inspired Lego Approach for Controlling the Wettability of Surfaces with Colorless Coatings. *Biomimetics* **2023**, *8*, 3.

2. Anraku Y., Kuwahara H., Fukusato Y., Mizoguchi A., Ishii T., Nika K., Matsumoto Y., Toh K., Miyata K., Uchida S., et al. Glycaemic Control Boosts Glucosylated Nanocarrier Crossing the BBB into the Brain. *Nat. Commun*. **2017**, 8:1001.

S4. ^{1}H and ^{13}C NMR spectra of new compounds

^{1}H, ^{13}C NMR and IR spectra of compound **19**

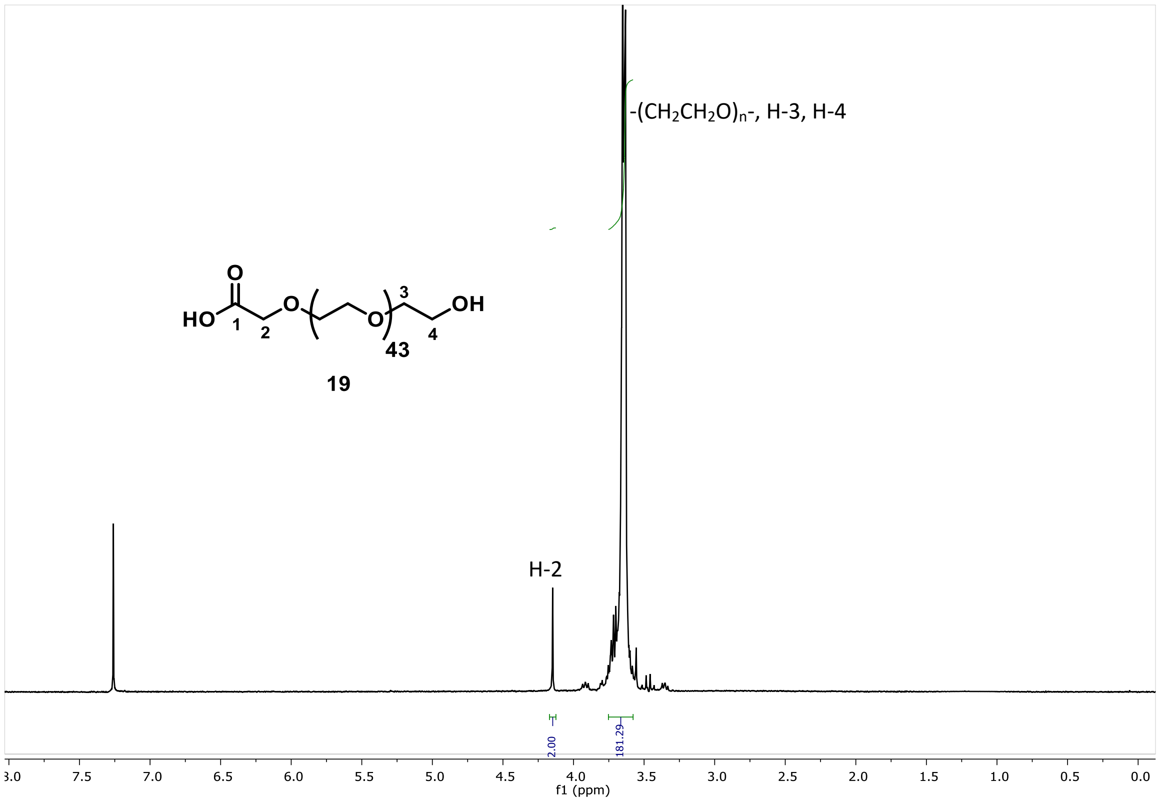

^{1}H NMR (400 MHz, $CDCl_3$)

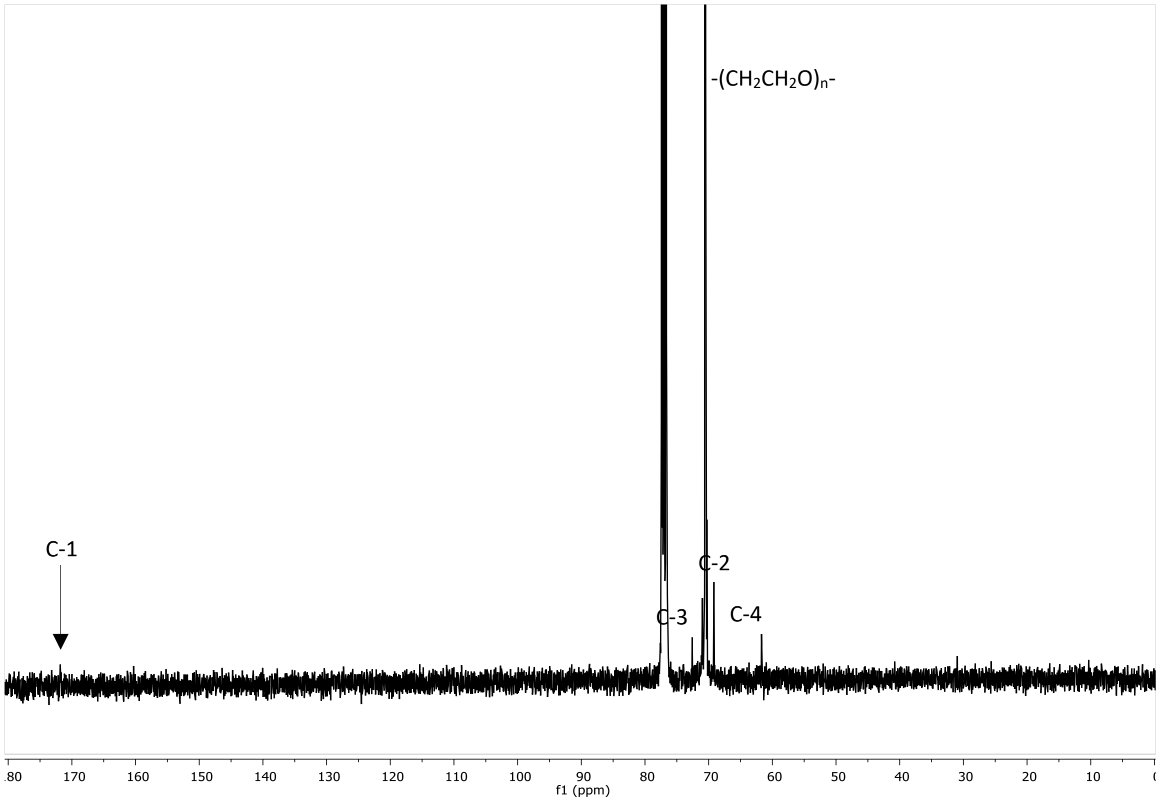

^{13}C NMR (100.6 MHz, $CDCl_3$)

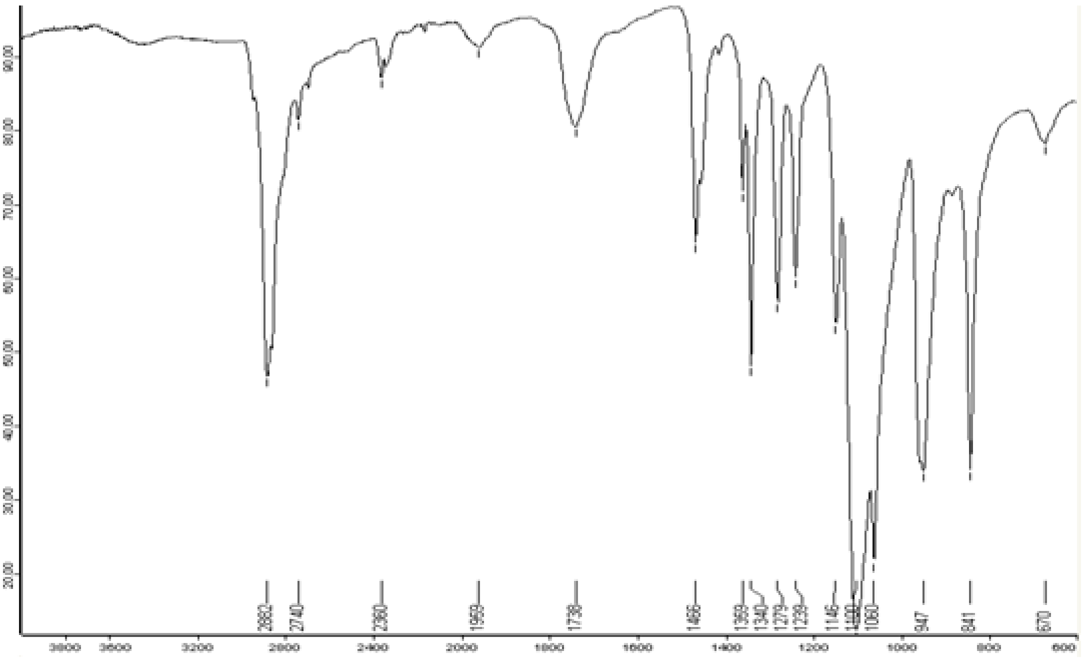
90,00
80,00
70,00
60,00
50,00
40,00
30,00
20,00
2862
2740
2360
1959
1738
1466
1369
1340
1279
1239
1146
1060
947
841
670
3800
3600
3200
2800
2400
2000
1800
1600
1400
1200
1000
800
600

IR (ATR)

^1H, ^{13}C NMR and IR spectra of compound **9**

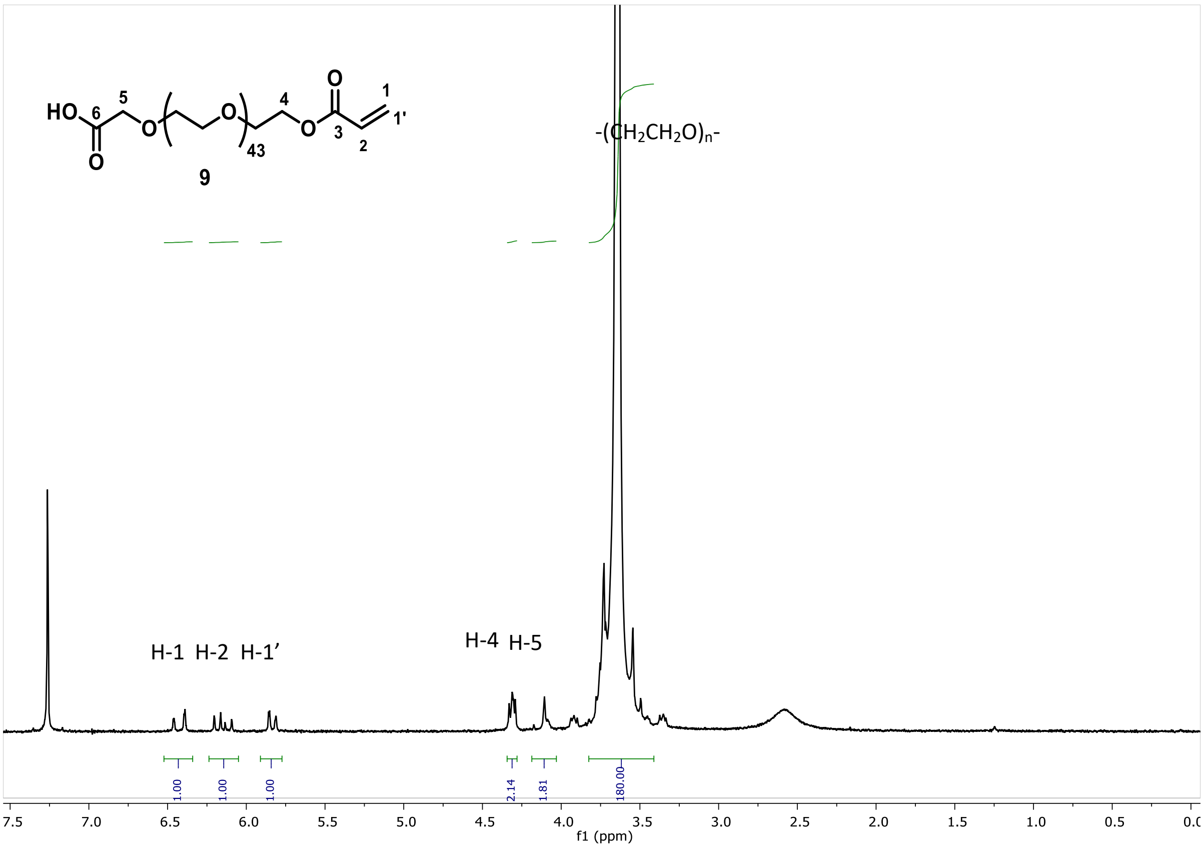

^1H NMR (400 MHz, $CDCl_3$)

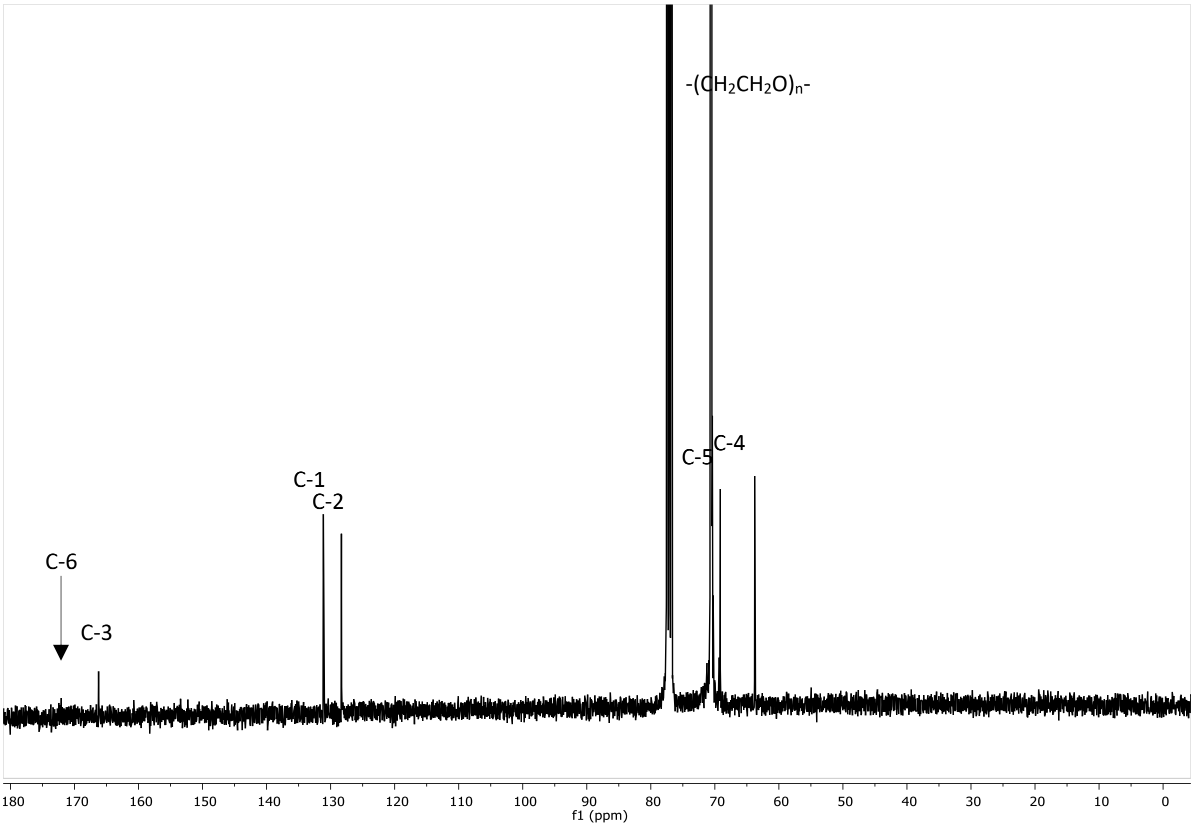

^{13}C NMR (100.6 MHz, $CDCl_3$)

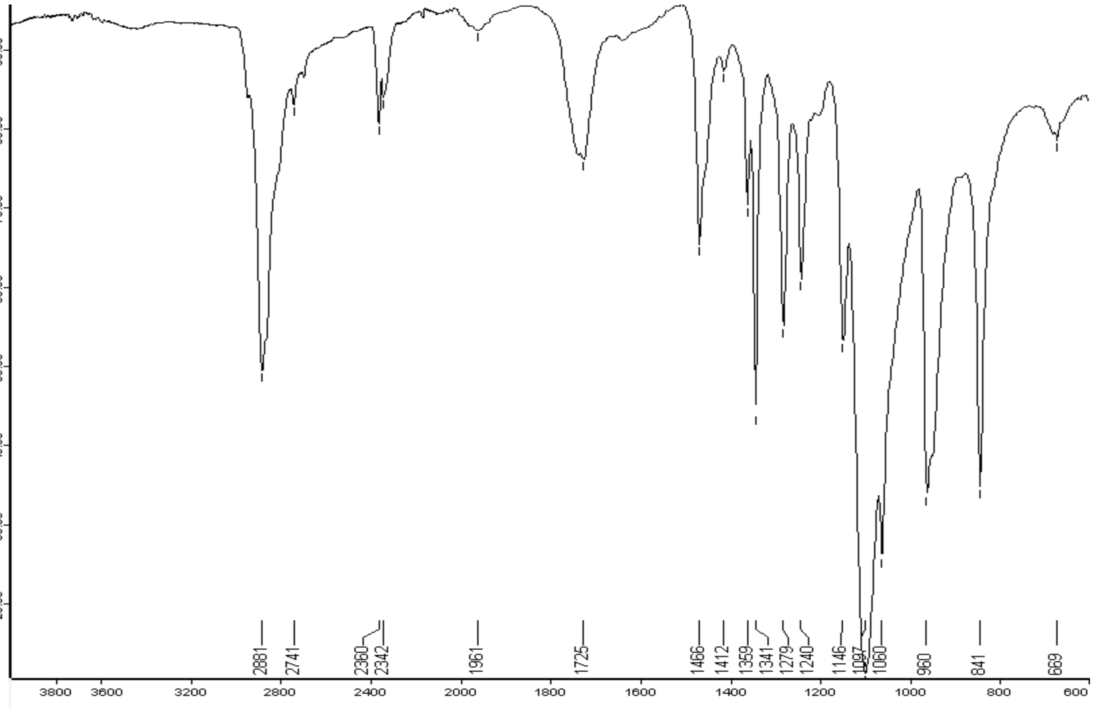
90.00
80.00
70.00
60.00
50.00
40.00
30.00
20.00
2881
2741
2360
2342
1961
1725
1466
1412
1359
1341
1279
1240
1146
1097
1060
960
841
669
3800
3600
3200
2800
2400
2000
1800
1600
1400
1200
1000
800
600

IR (ATR)

^{1}H, ^{13}C NMR and IR spectra of compound **10**

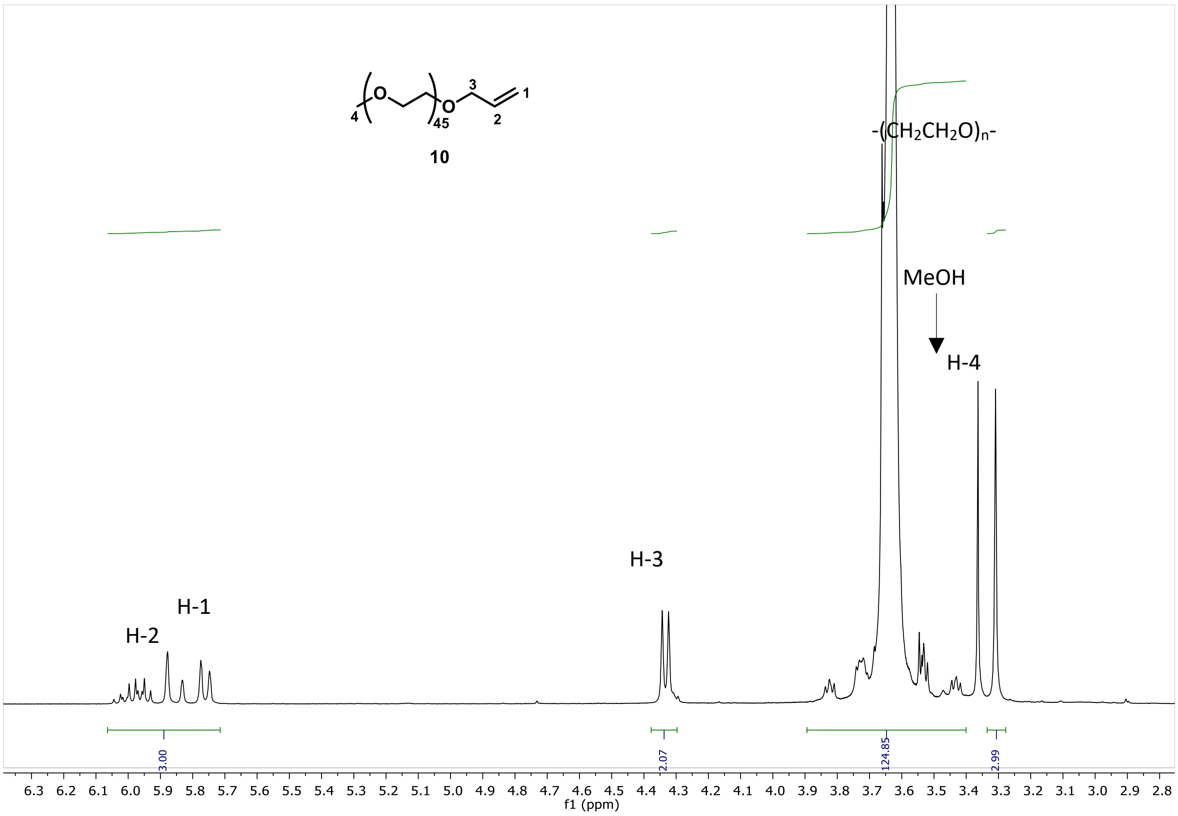

^{1}H NMR (360 MHz, $CDCl_3$)

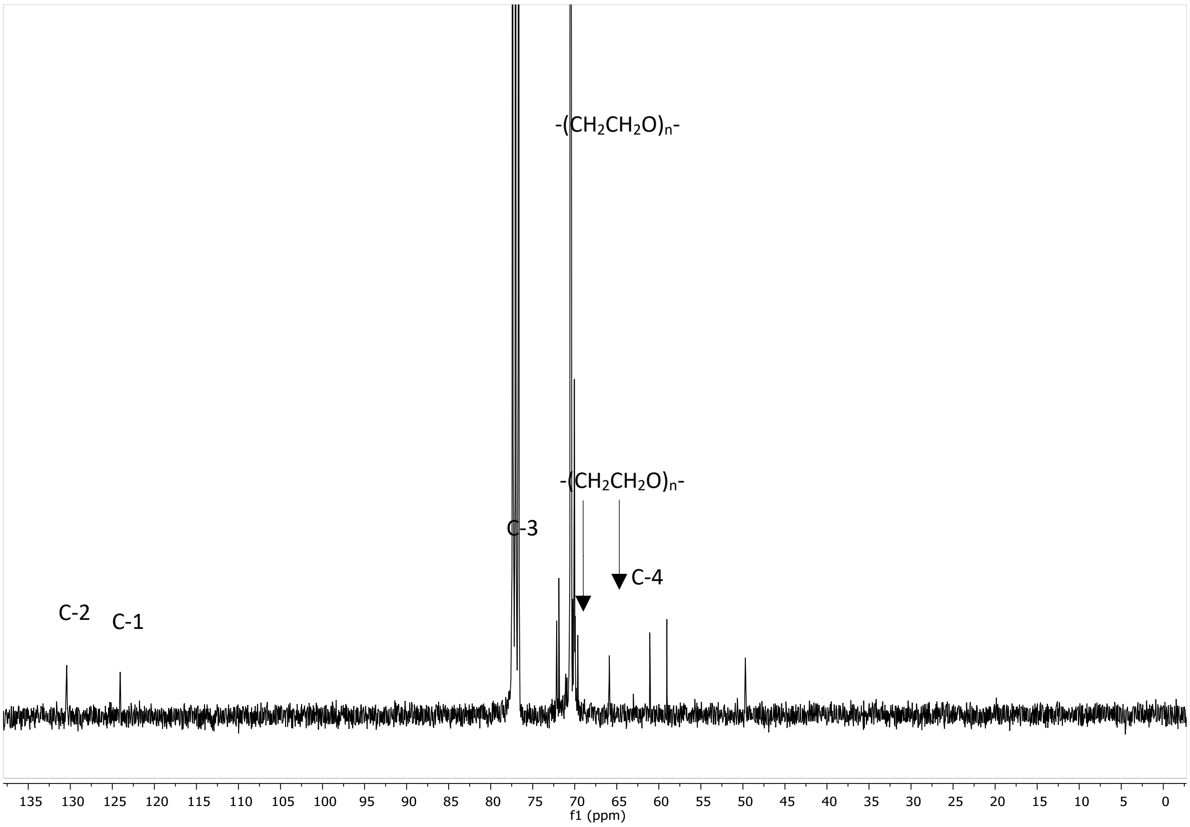

^{13}C NMR (90.5 MHz, $CDCl_3$)

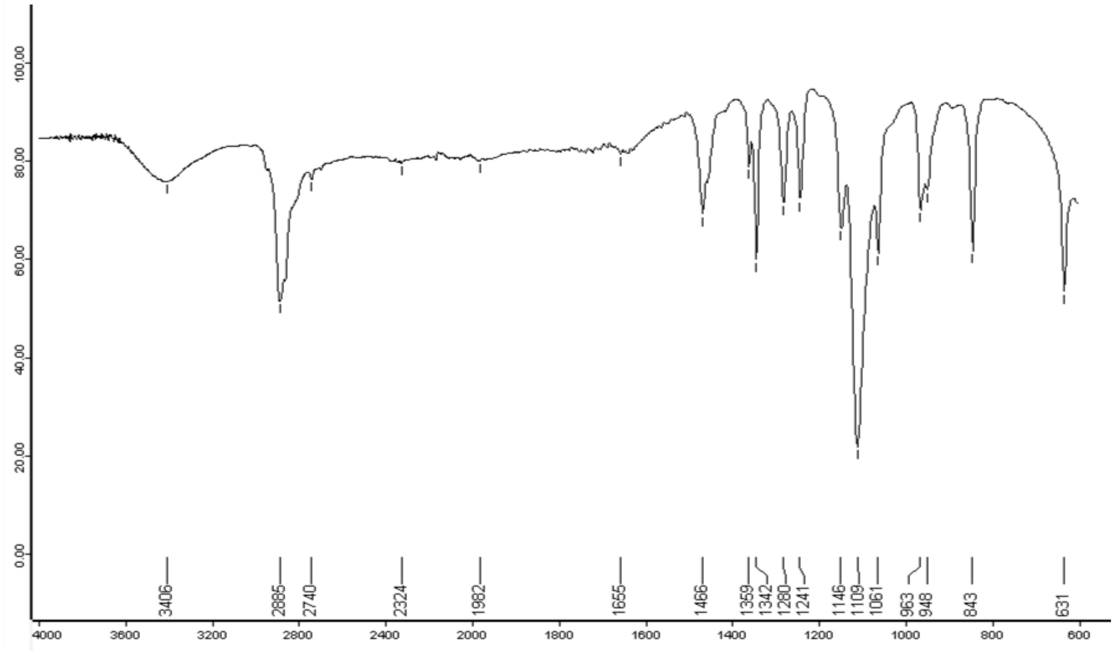

100,00
80,00
60,00
40,00
20,00
0,00
3406
2885
2740
2324
1982
1655
1466
1359
1342
1280
1241
1146
1109
1061
963
948
843
631
4000
3600
3200
2800
2400
2000
1800
1600
1400
1200
1000
800
600

IR (ATR)

^{1}H, ^{13}C NMR and IR spectra of compound **1**

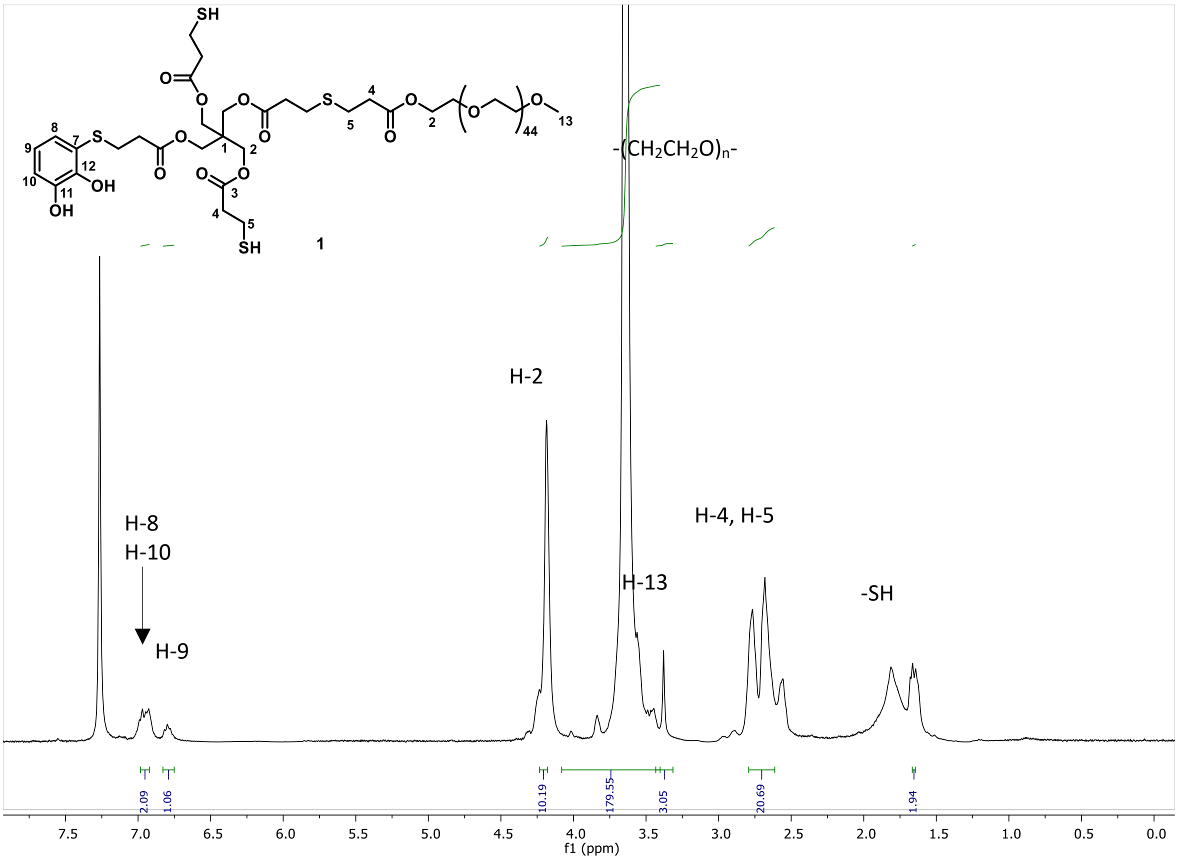

^{1}H NMR (500 MHz, $CDCl_3$)

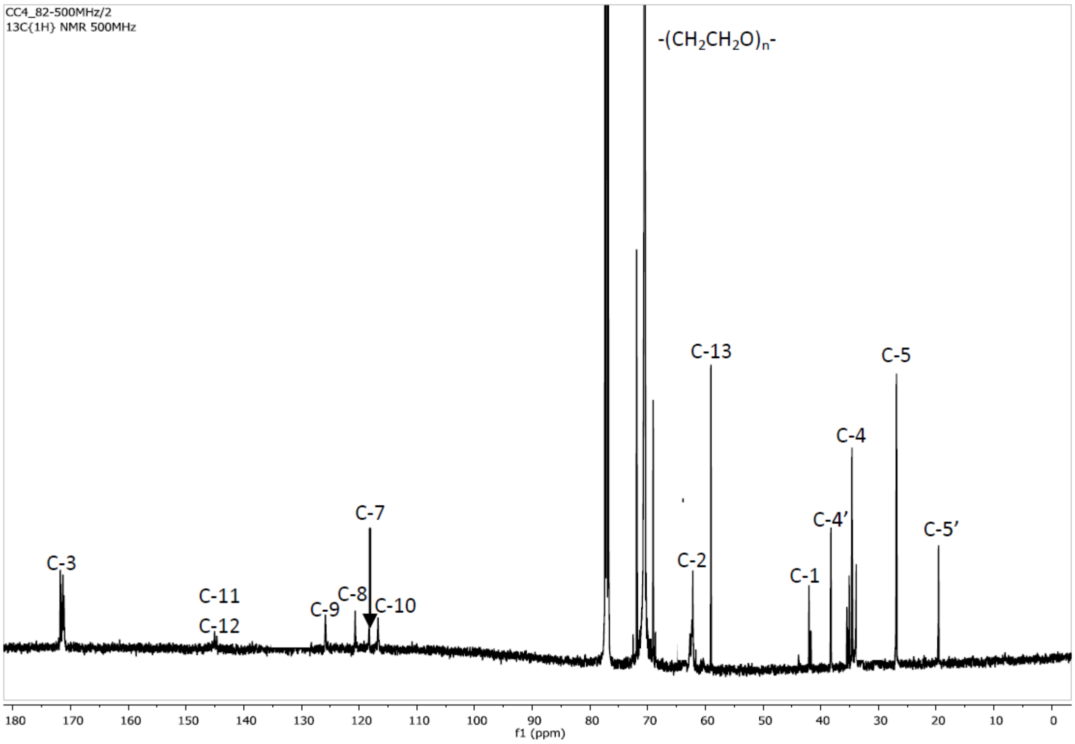

^{13}C NMR (125.8 MHz, $CDCl_3$)

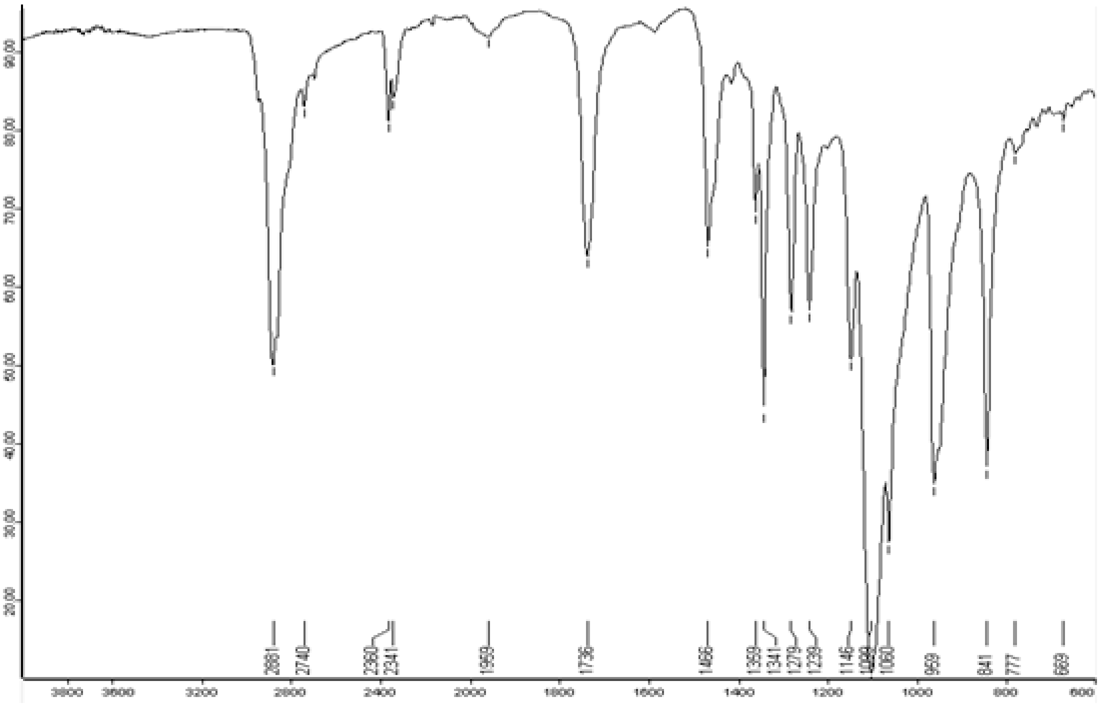
2861
2740
2360
2341
1959
1736
1466
1359
1341
1279
1239
1146
1060
959
841
777
669

IR (ATR)

^1H, ^{13}C NMR and IR spectra of compound **2**

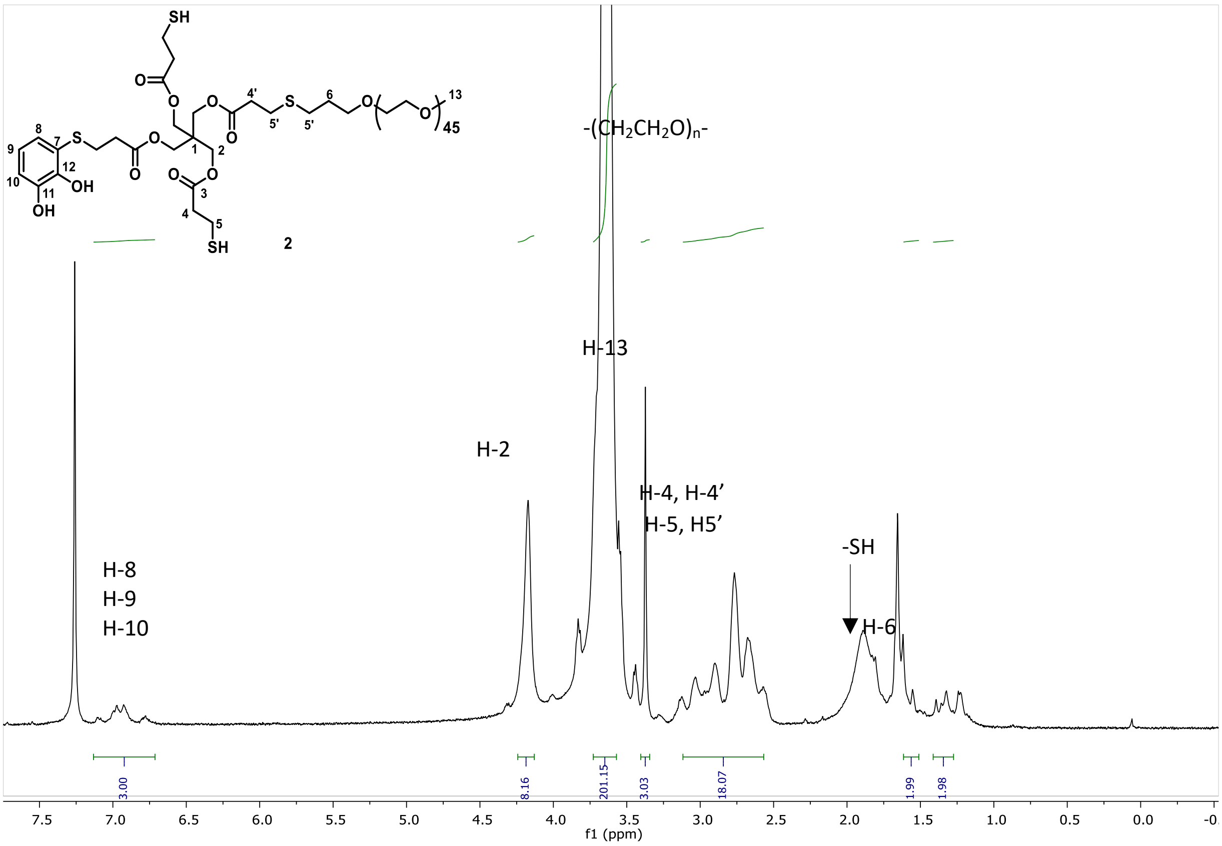

^1H NMR (360 MHz, $CDCl_3$)

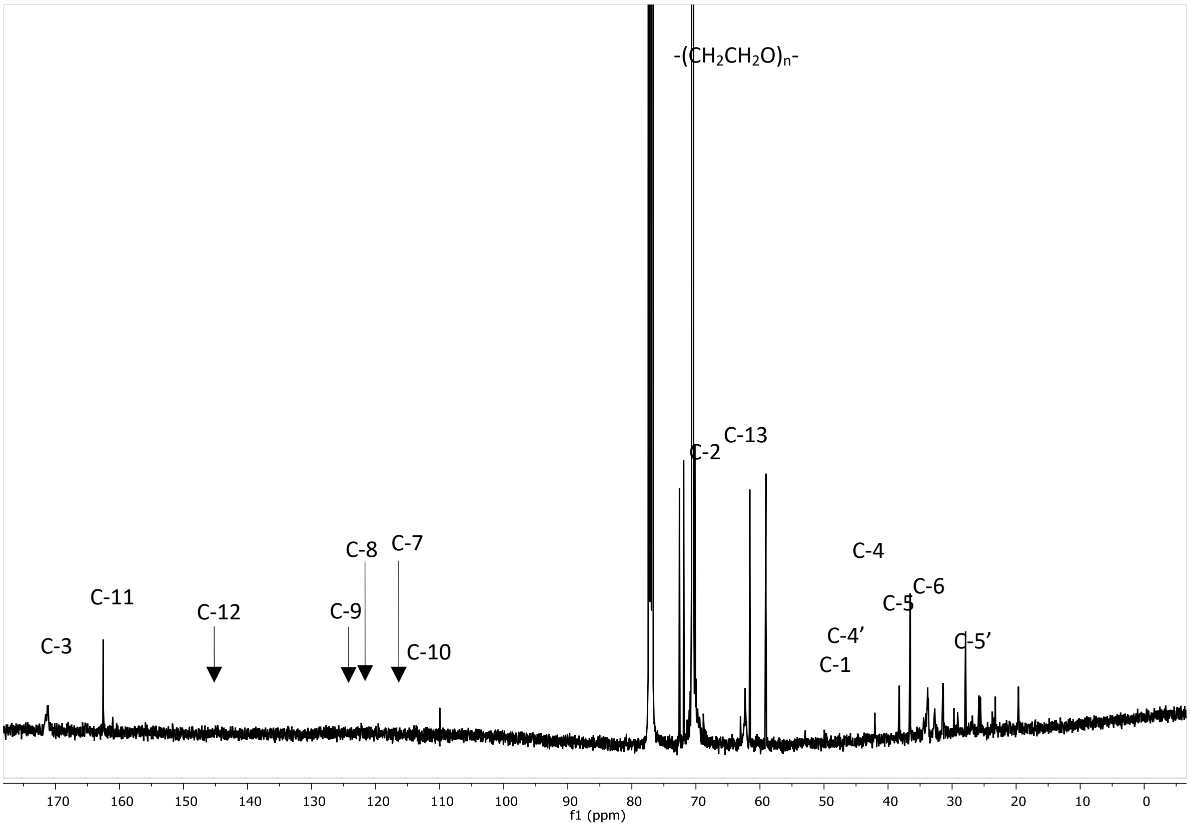

^{13}C NMR (125.8 MHz, $CDCl_3$)

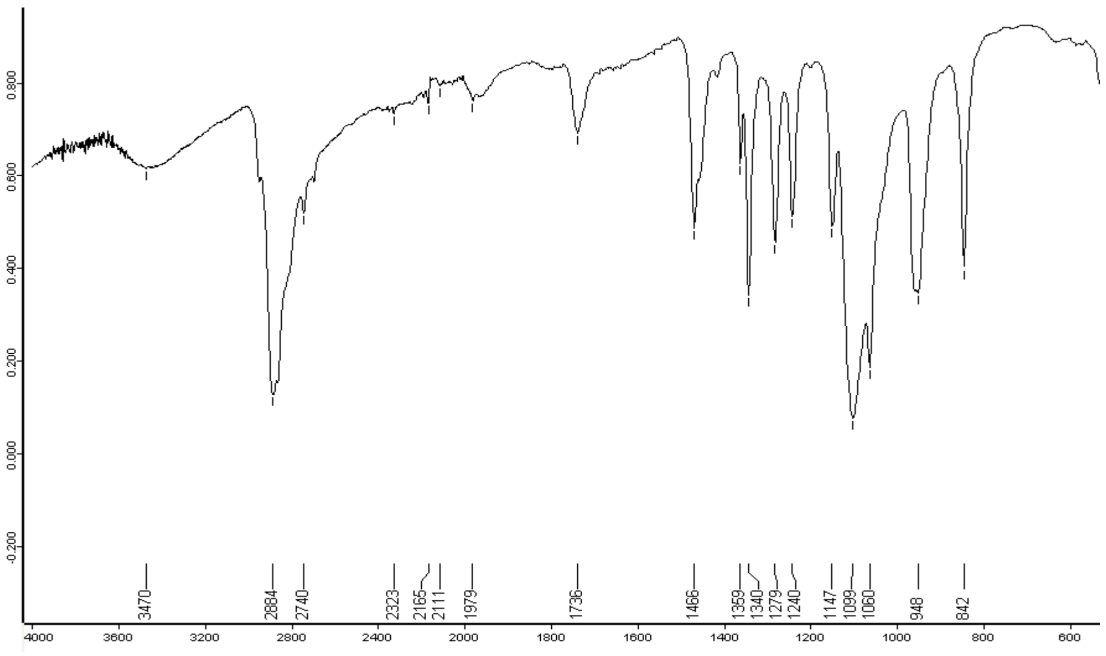
0.800
0.600
0.400
0.200
0.000
-0.200
3470
2884
2740
2323
2165
2111
1979
1736
1466
1359
1340
1279
1240
1147
1099
1060
948
842
4000
3600
3200
2800
2400
2000
1800
1600
1400
1200
1000
800
600

IR (ATR)

^{1}H, ^{13}C NMR and IR spectra of compound **5**

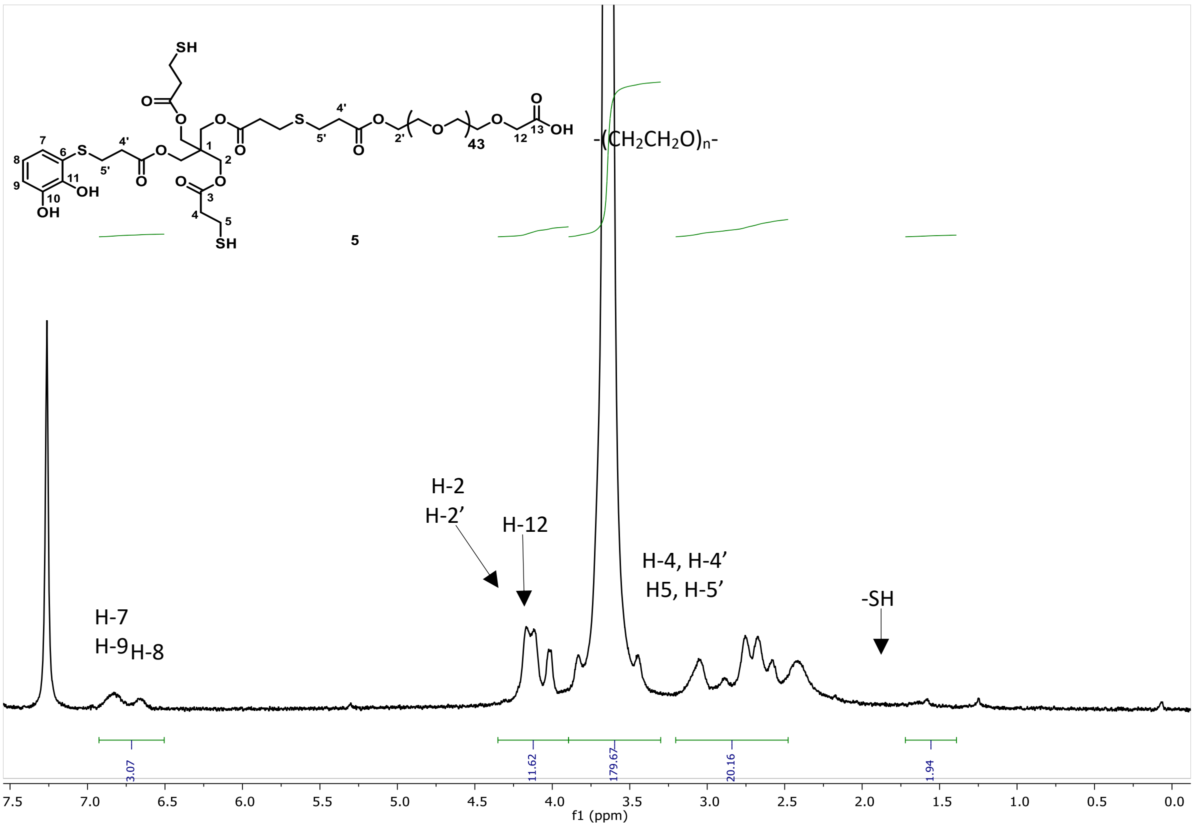

^{1}H NMR (360 MHz, $CDCl_3$)

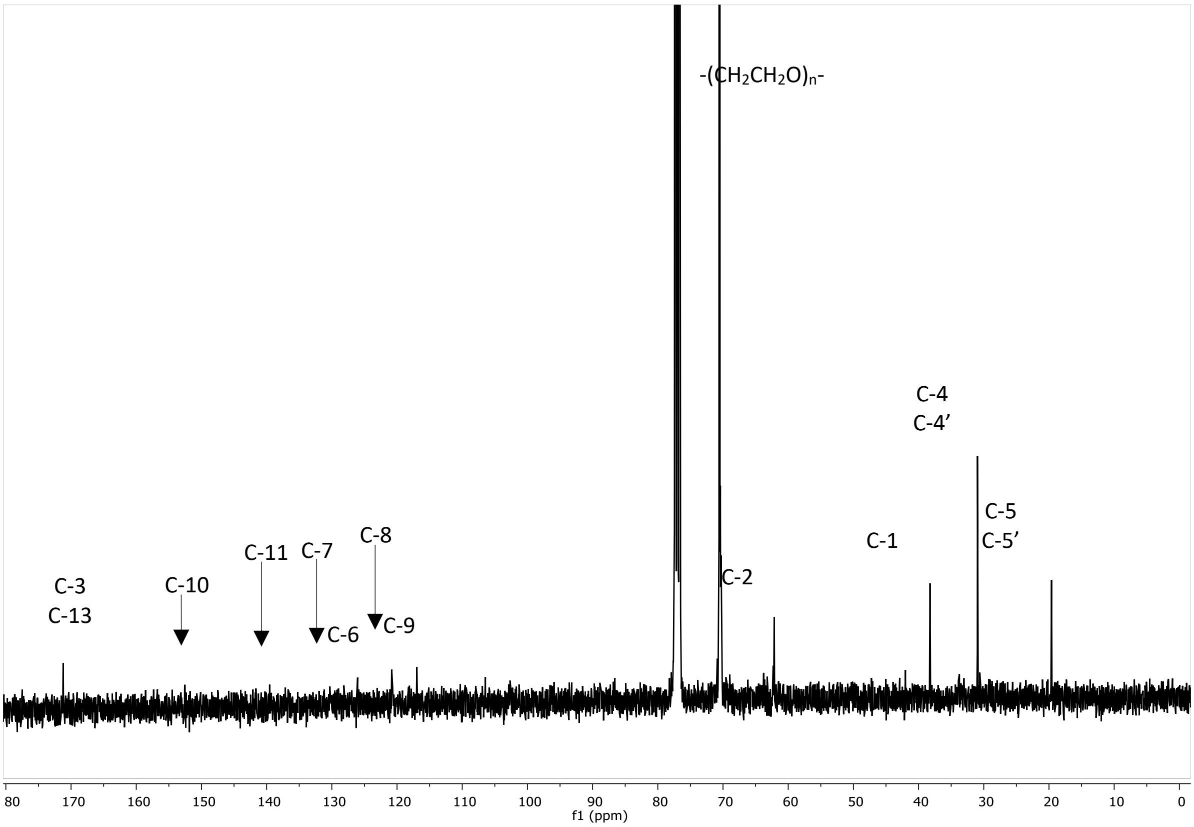

^{13}C NMR (90.5 MHz, $CDCl_3$)

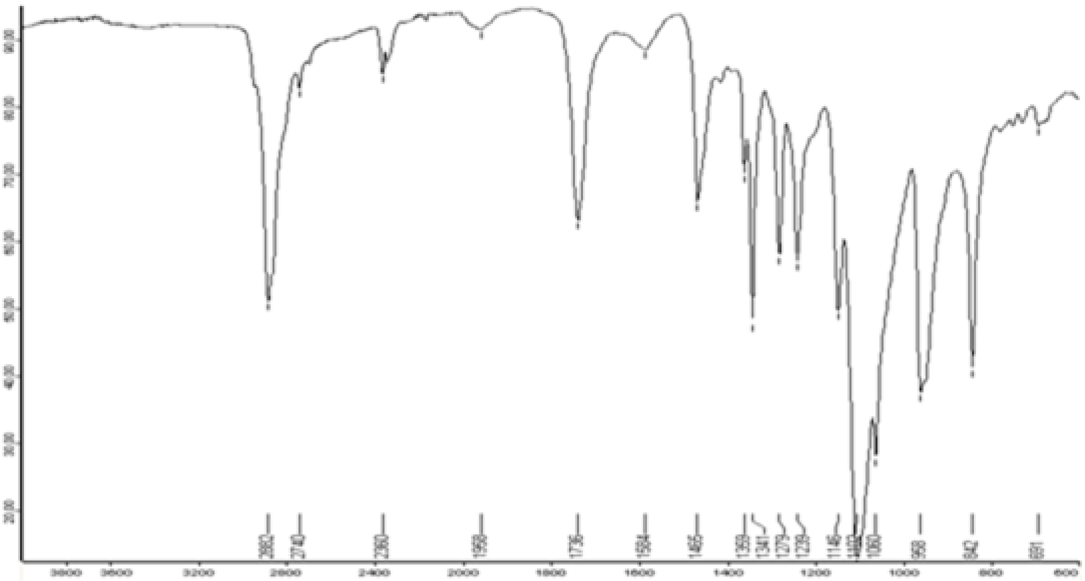
2863
2740
2360
1959
1736
1584
1465
1359
1341
1279
1239
1146
1060
958
842
691
3800
3600
3200
2800
2400
2000
1800
1600
1400
1200
1000
800
600

IR (ATR)

^{1}H, ^{13}C NMR and IR spectra of compound **15**

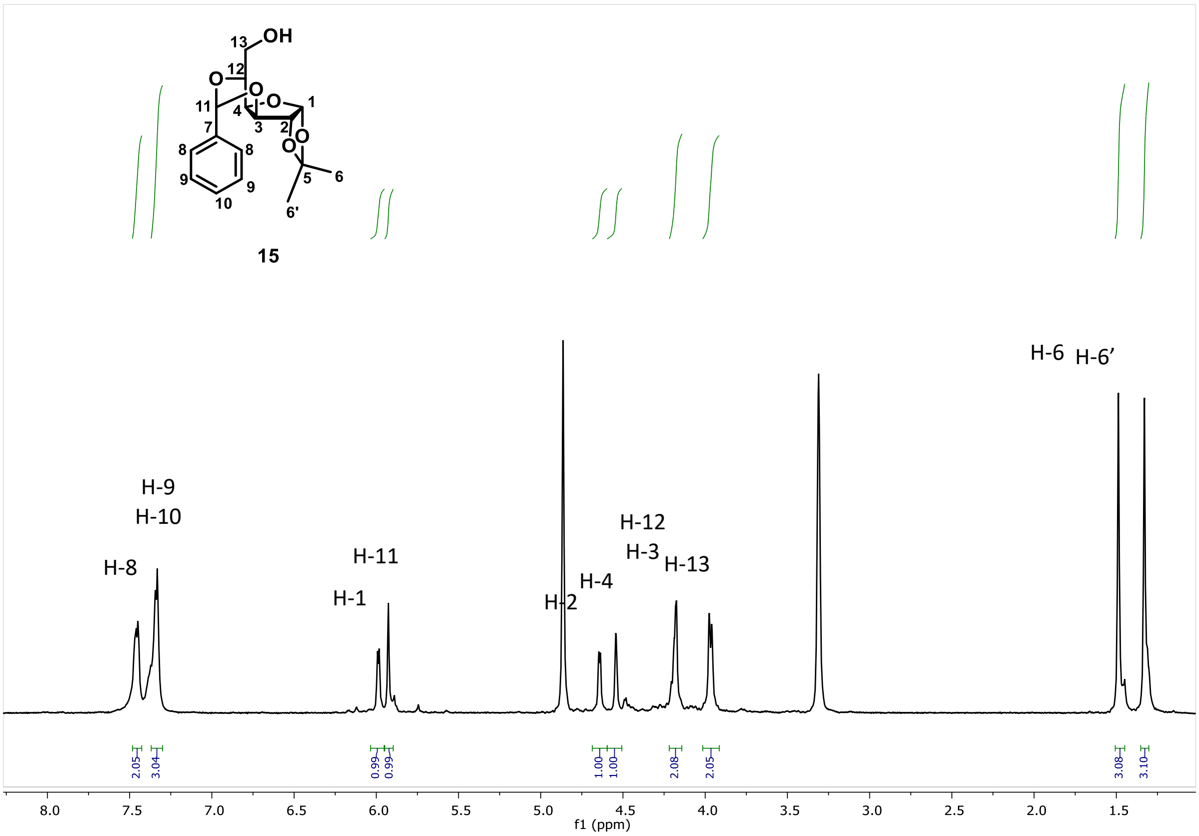

^{1}H NMR (360 MHz, CD_3OD)

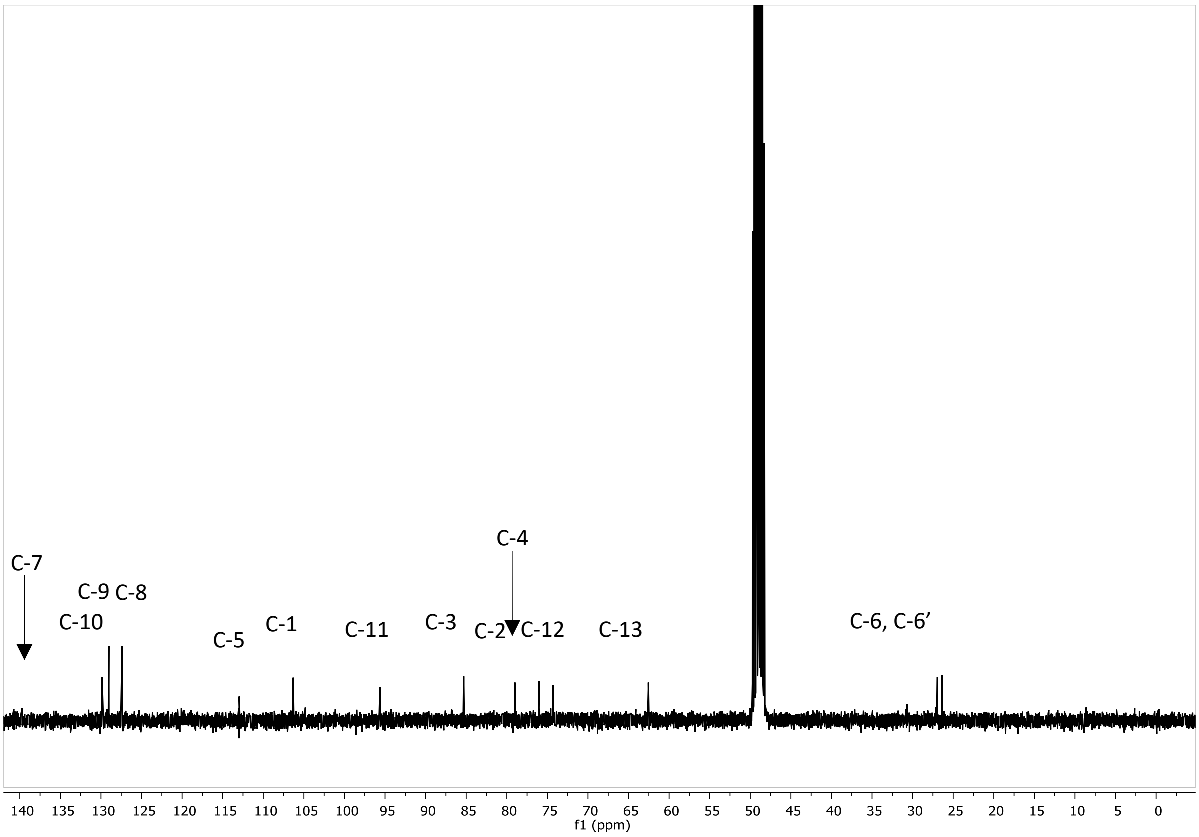

^{13}C NMR (90.5 MHz, CD_3OD)

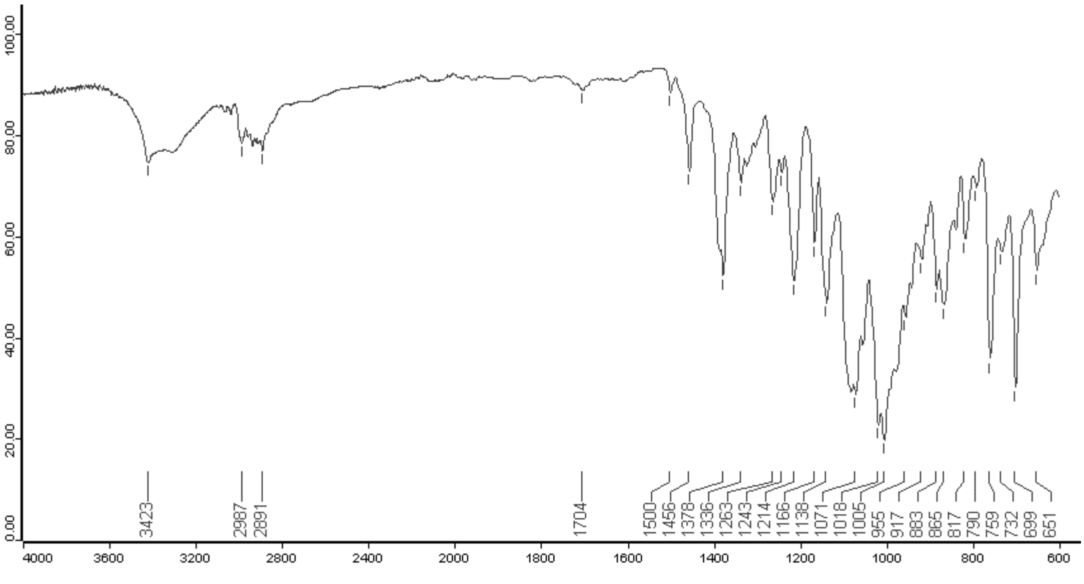
100.00
80.00
60.00
40.00
20.00
0.00
3423
2987
2891
1704
1500
1456
1378
1336
1263
1243
1214
1166
1138
1071
1018
1005
955
917
883
865
817
790
759
732
699
651
4000
3600
3200
2800
2400
2000
1800
1600
1400
1200
1000
800
600

IR (ATR)

^{1}H, ^{13}C NMR and IR spectra of compound **16**

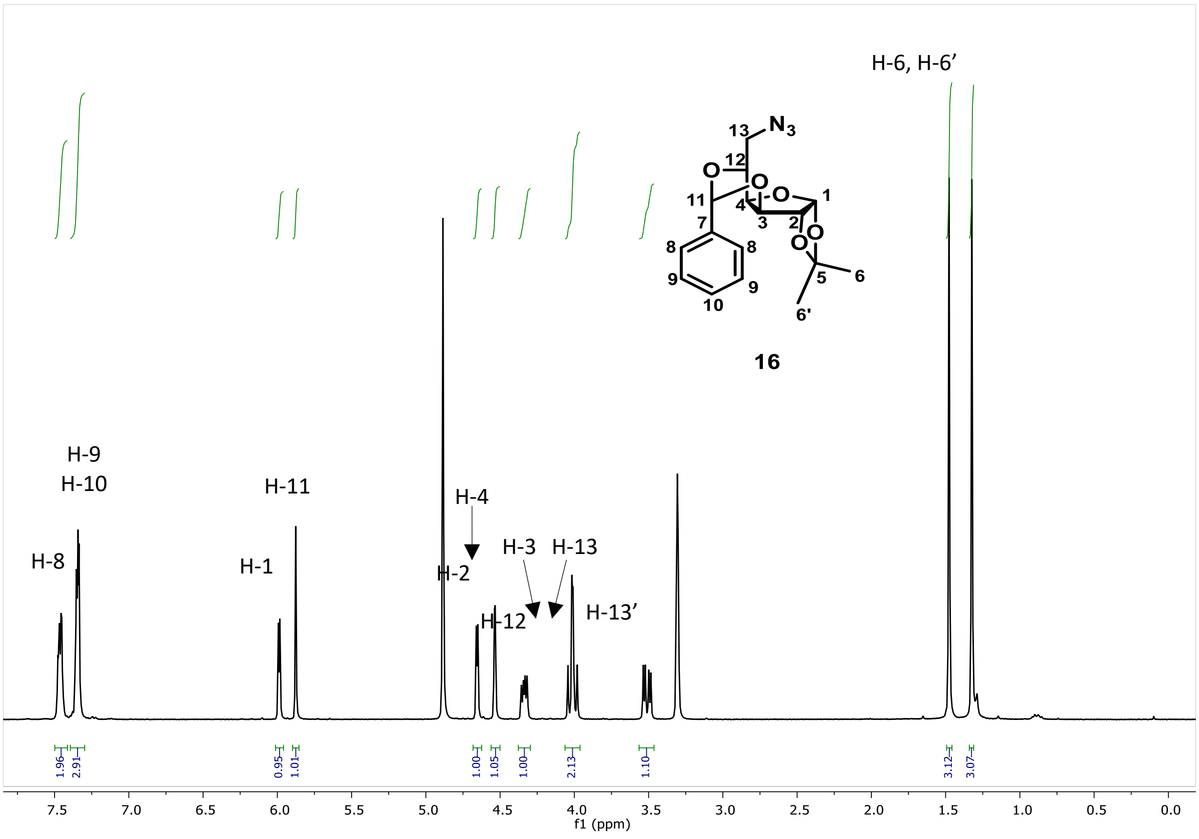

^{1}H NMR (360 MHz, CD_3OD)

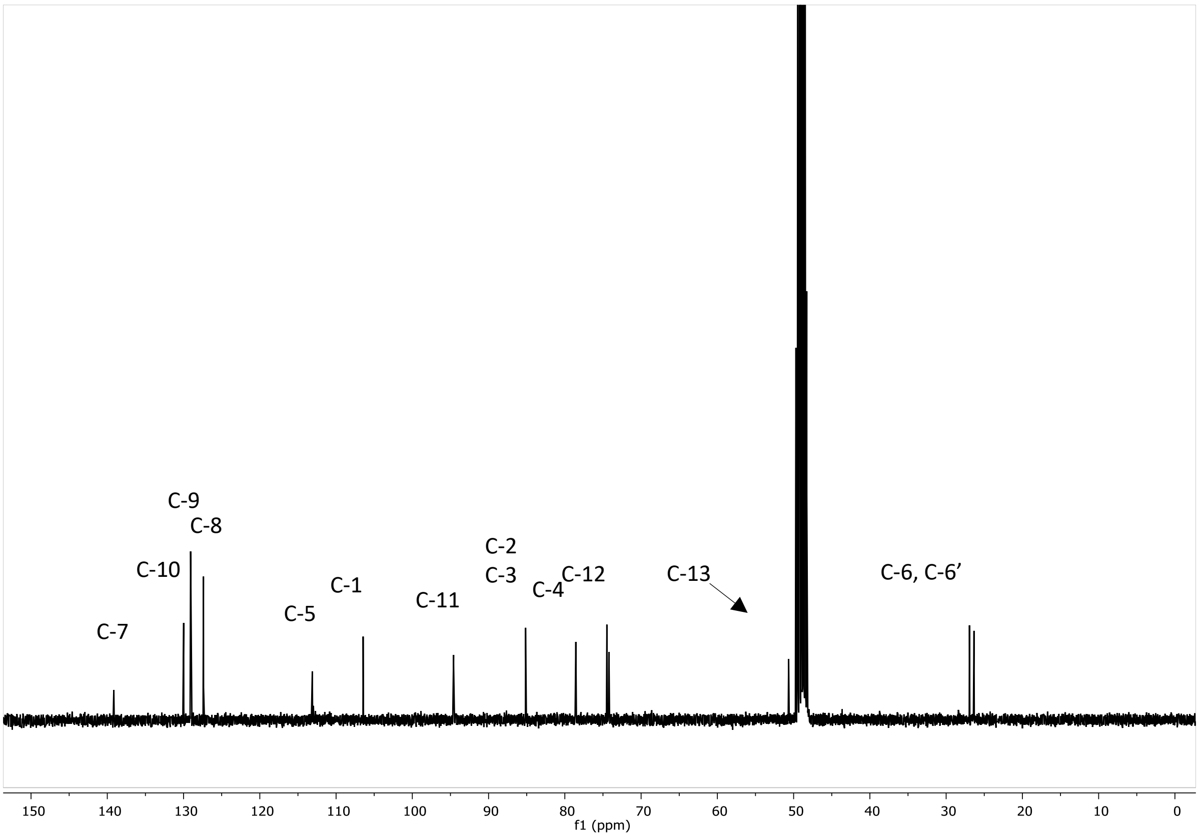

^{13}C NMR (90.5 MHz, CD_3OD)

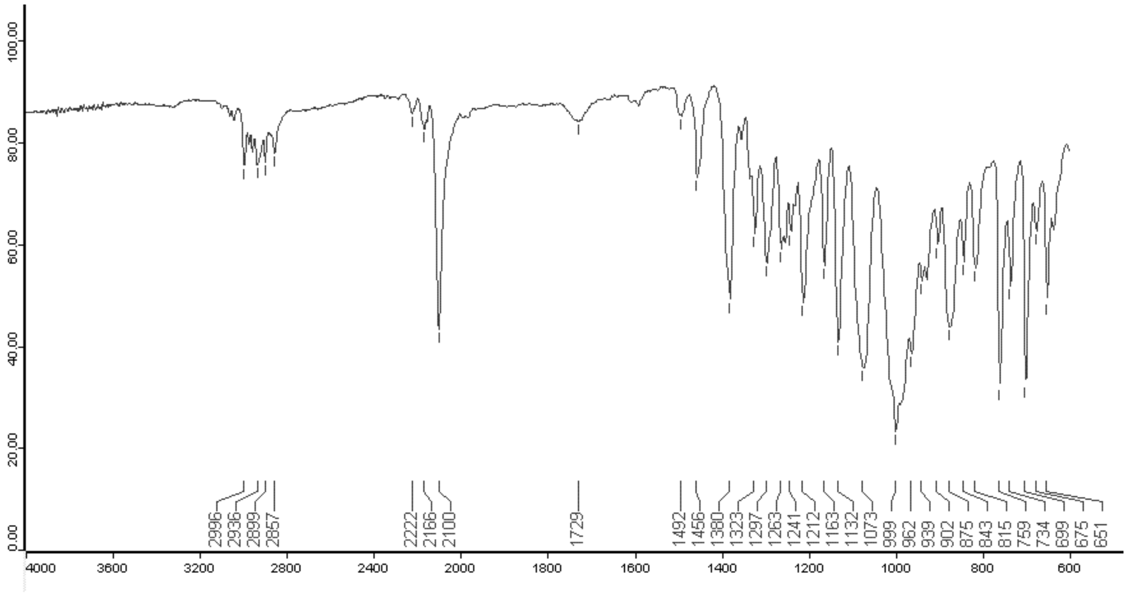
100.00
80.00
60.00
40.00
20.00
0.00
2996
2936
2899
2857
2222
2166
2100
1729
1492
1456
1380
1323
1297
1263
1241
1212
1163
1132
1073
999
962
939
902
875
843
815
759
734
699
675
651
4000
3600
3200
2800
2400
2000
1800
1600
1400
1200
1000
800
600

IR (ATR)

^{1}H, ^{13}C NMR and IR spectra of compound **12**

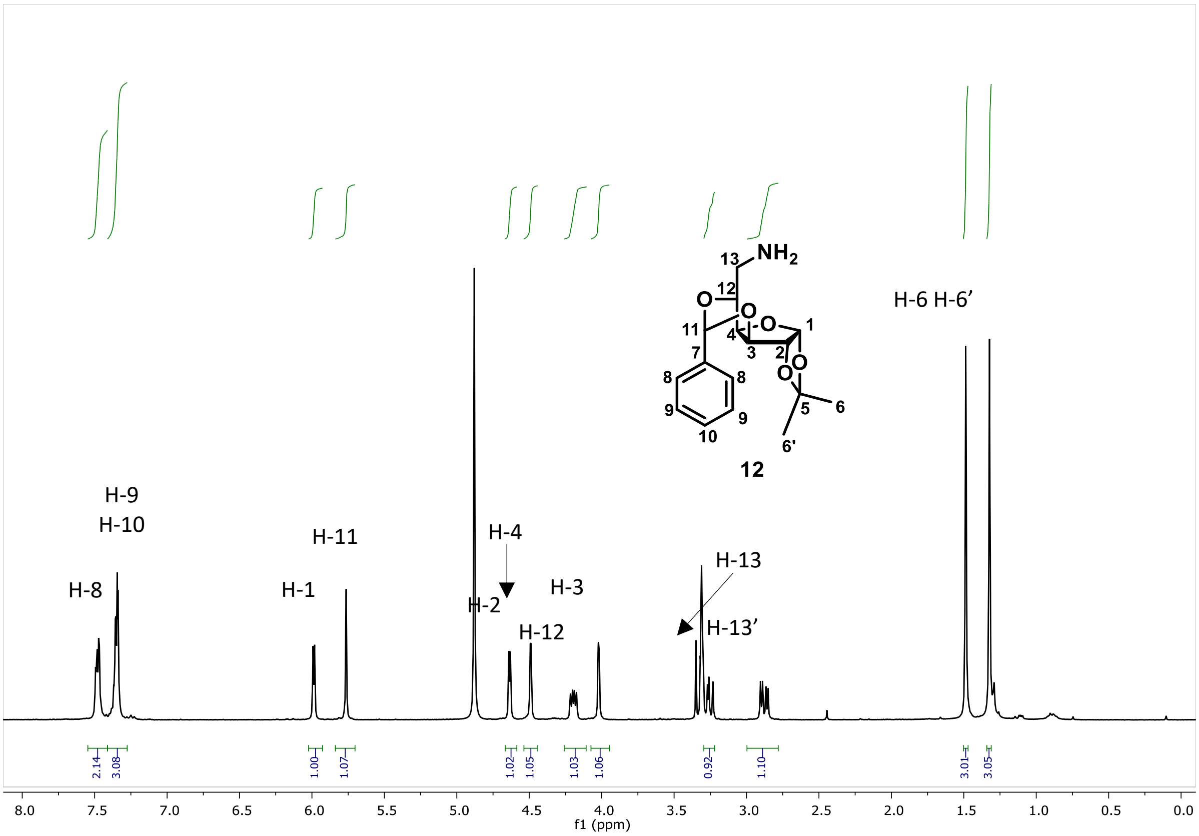

^{1}H NMR (400 MHz, CD_3OD)

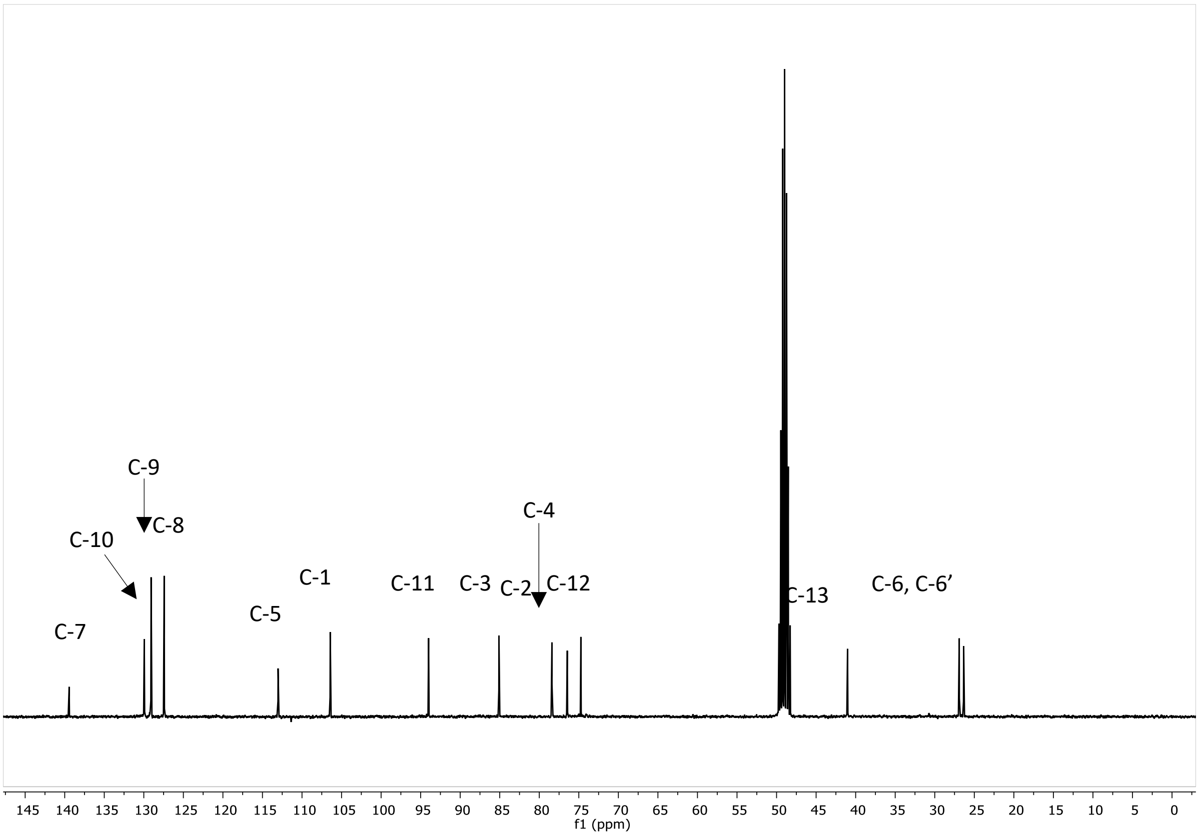

^{13}C NMR (100.6 MHz, CD_3OD)

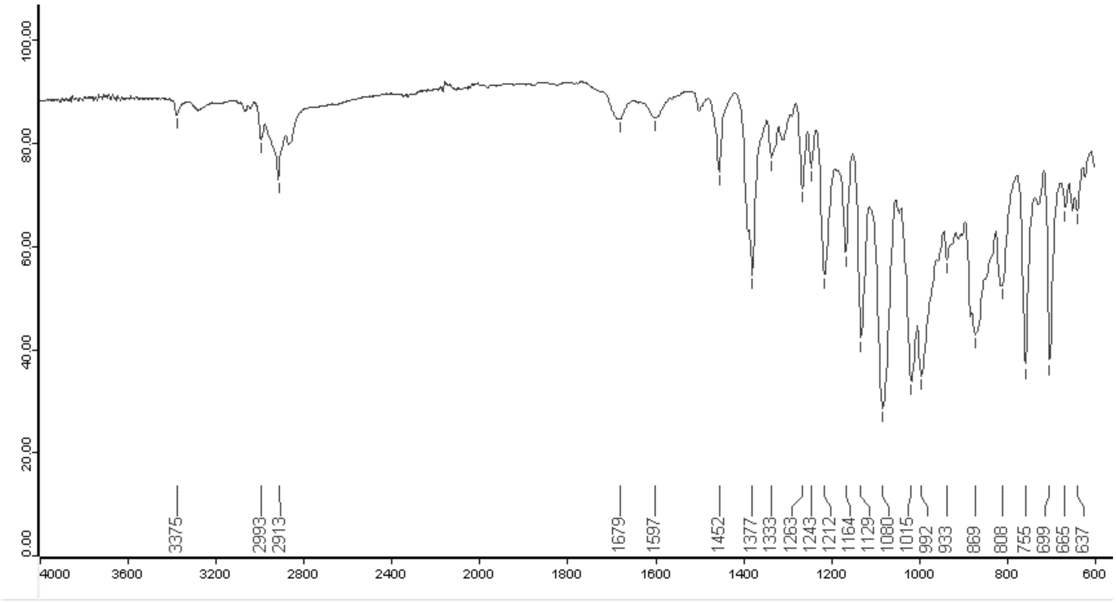
100,00
80,00
60,00
40,00
20,00
0,00
3375
2993
2913
1679
1597
1452
1377
1333
1263
1243
1212
1164
1129
1080
1015
992
933
869
808
755
699
665
637
4000
3600
3200
2800
2400
2000
1800
1600
1400
1200
1000
800
600

IR (ATR)

^{1}H and ^{13}C NMR spectra of compound **17**

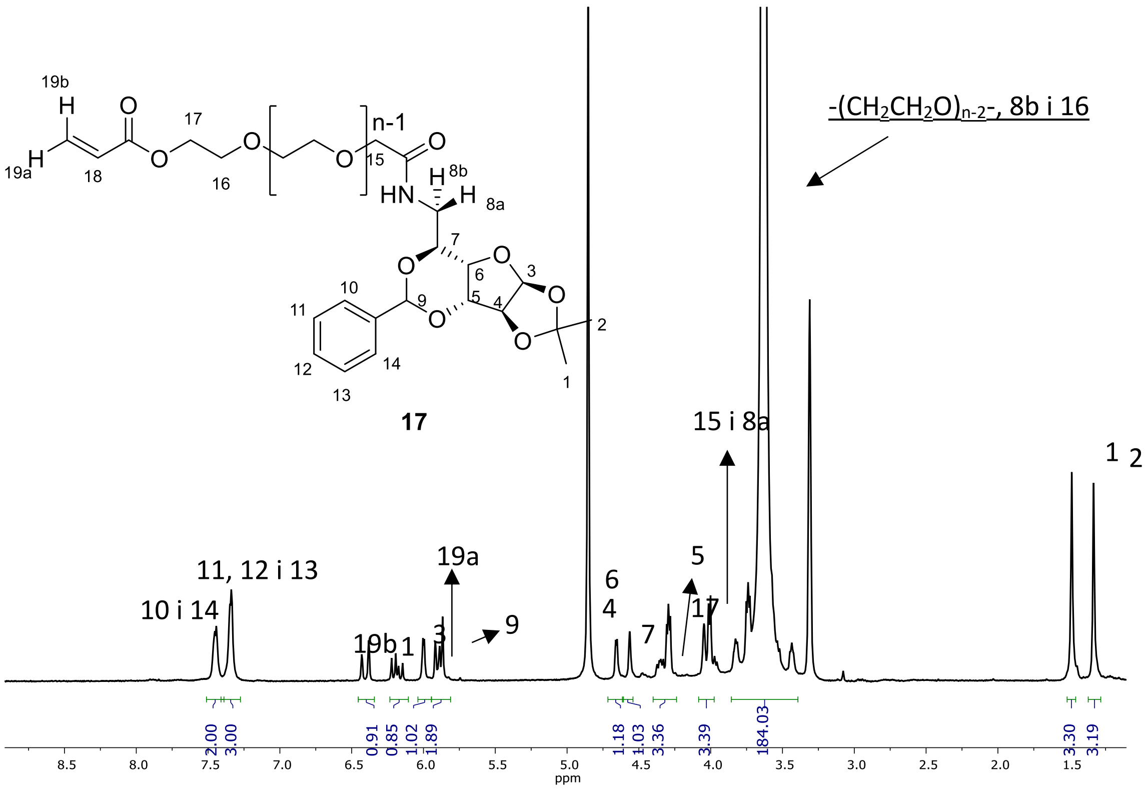

^{1}**H NMR** (360 MHz, CD_3OD)

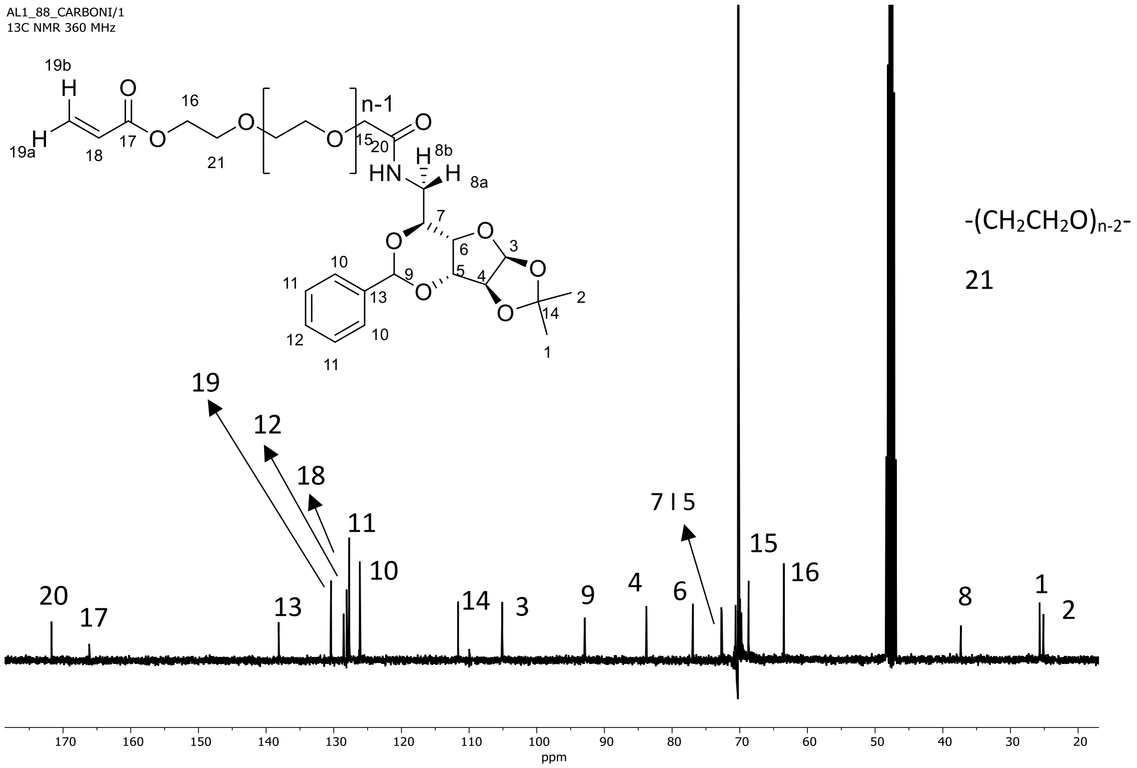

^{13}**C NMR** (360 MHz, CD_3OD)

^{1}H and spectrum of compound **20**

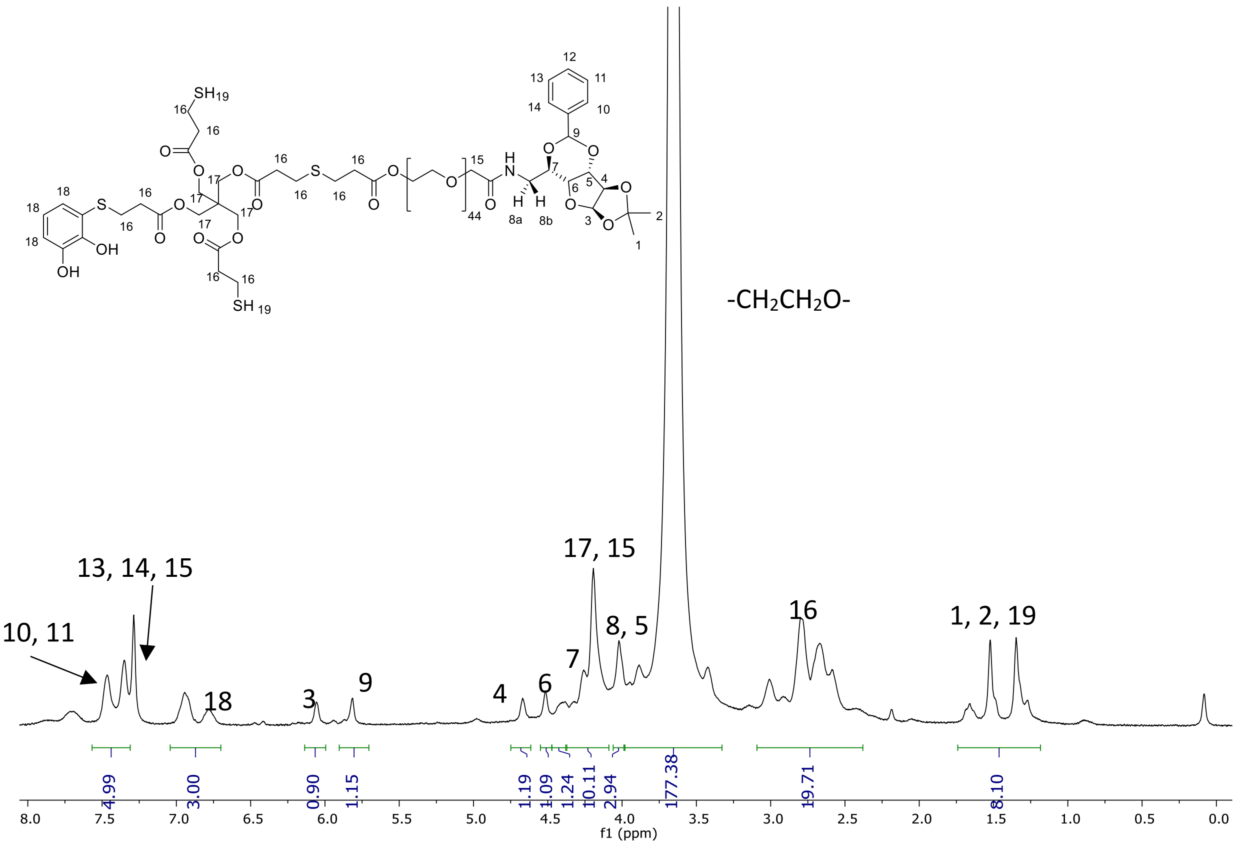

^{1}H NMR (360 MHz, $CDCl_3$

^{1}H, ^{13}C NMR and IR spectra of compound **13**

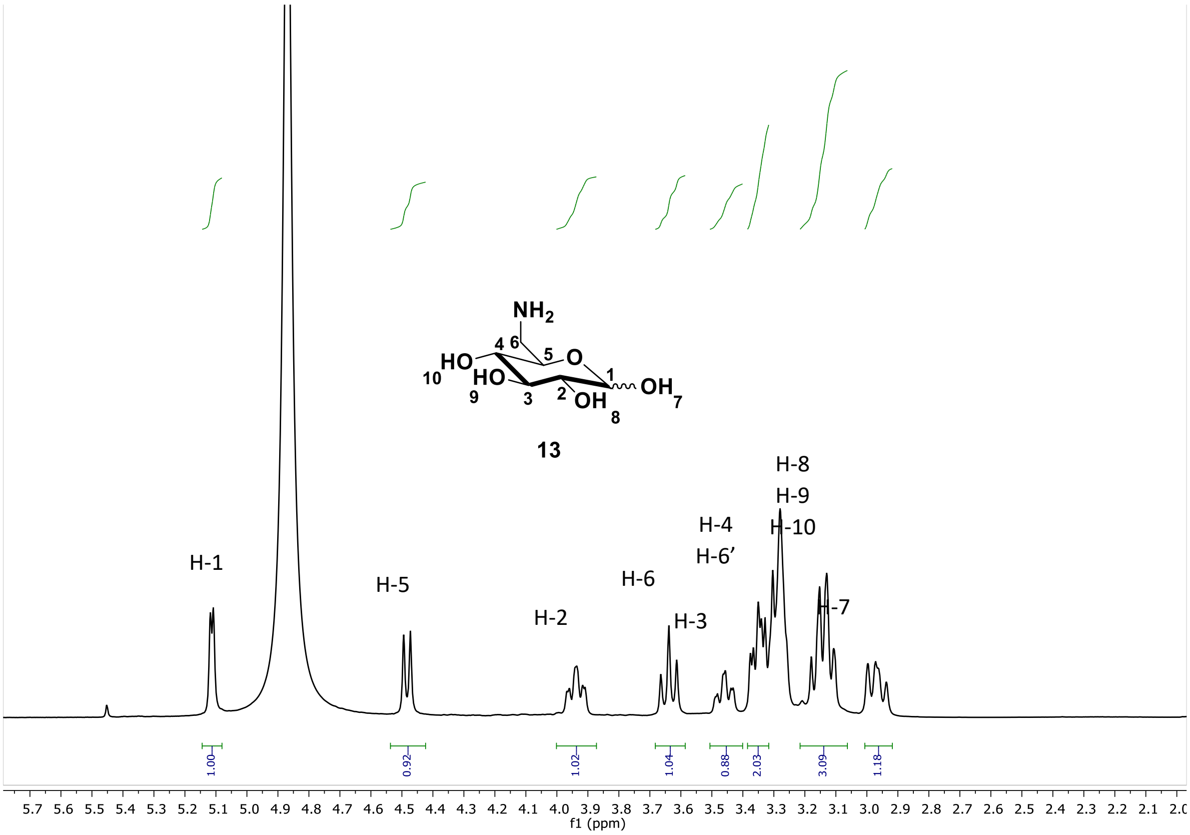

^{1}H NMR (360 MHz, CD_3OD)

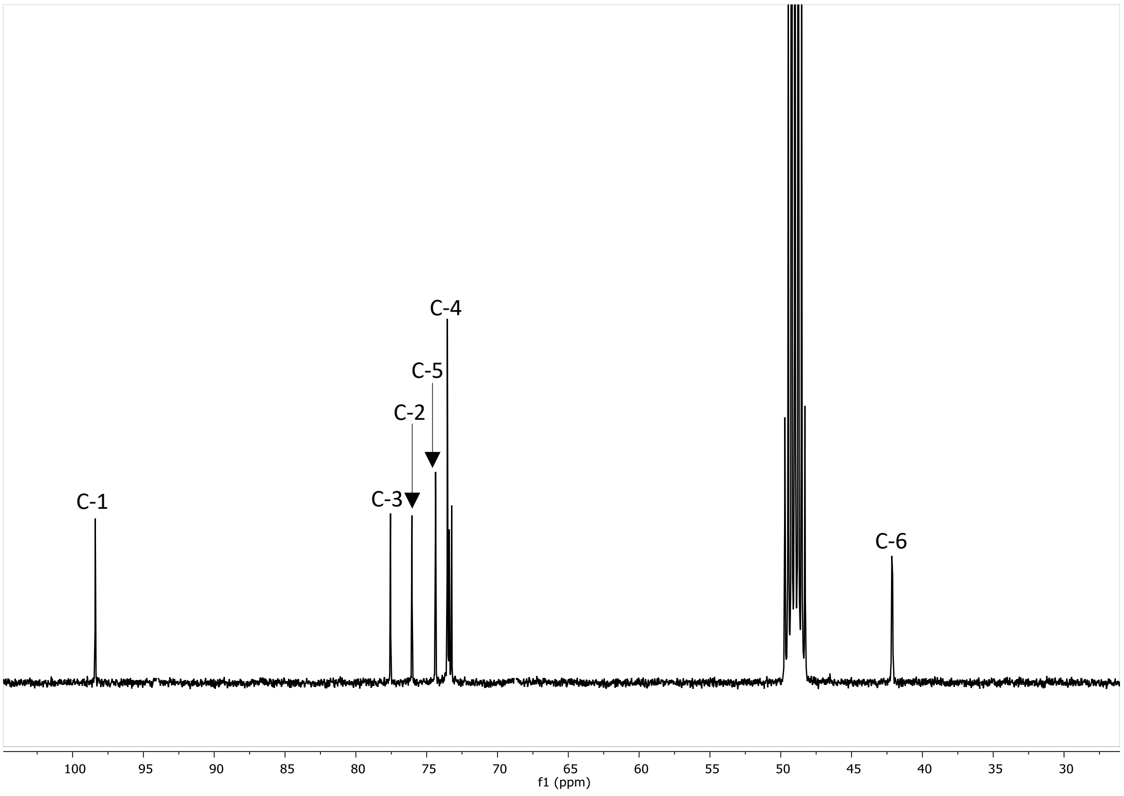

^{13}C NMR (90.5 MHz, CD_3OD)

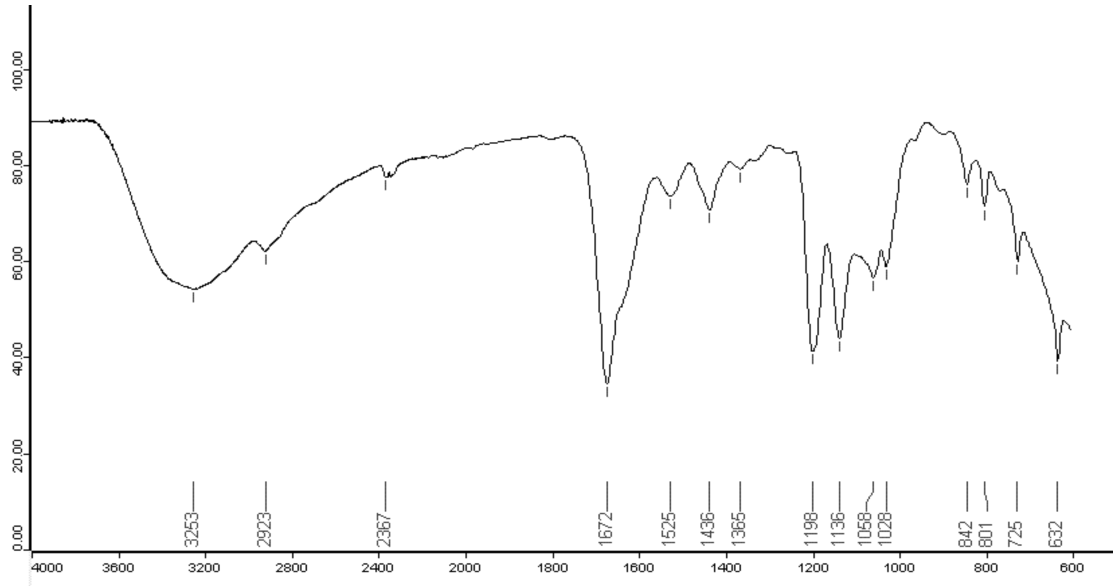
100,00
80,00
60,00
40,00
20,00
0,00
3253
2923
2367
1672
1525
1436
1365
1198
1136
1058
1028
842
801
725
632
4000
3600
3200
2800
2400
2000
1800
1600
1400
1200
1000
800
600

IR (ATR)

S5. 1H NMR and IR spectra of oligomers and copolimers

^{1}H NMR and IR spectra of oligomer **P1**

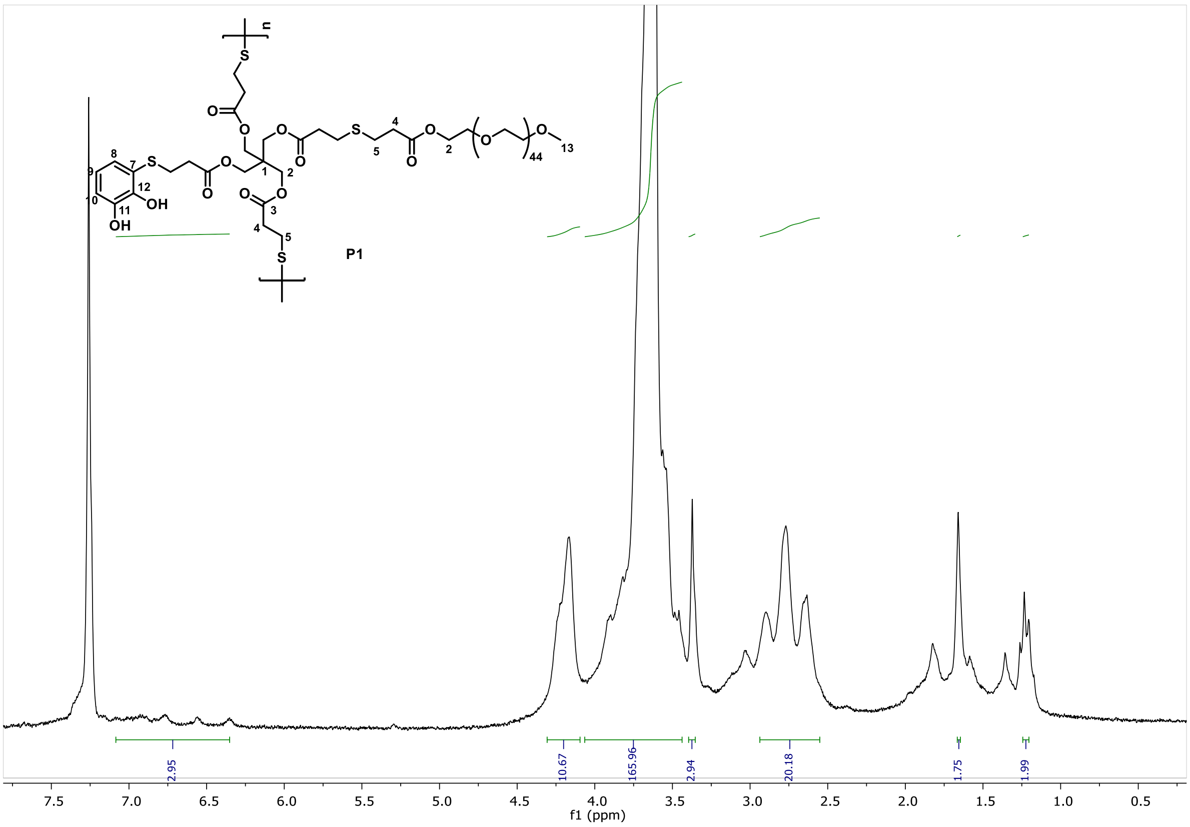

^{1}H NMR (400 MHz, $CDCl_3$)

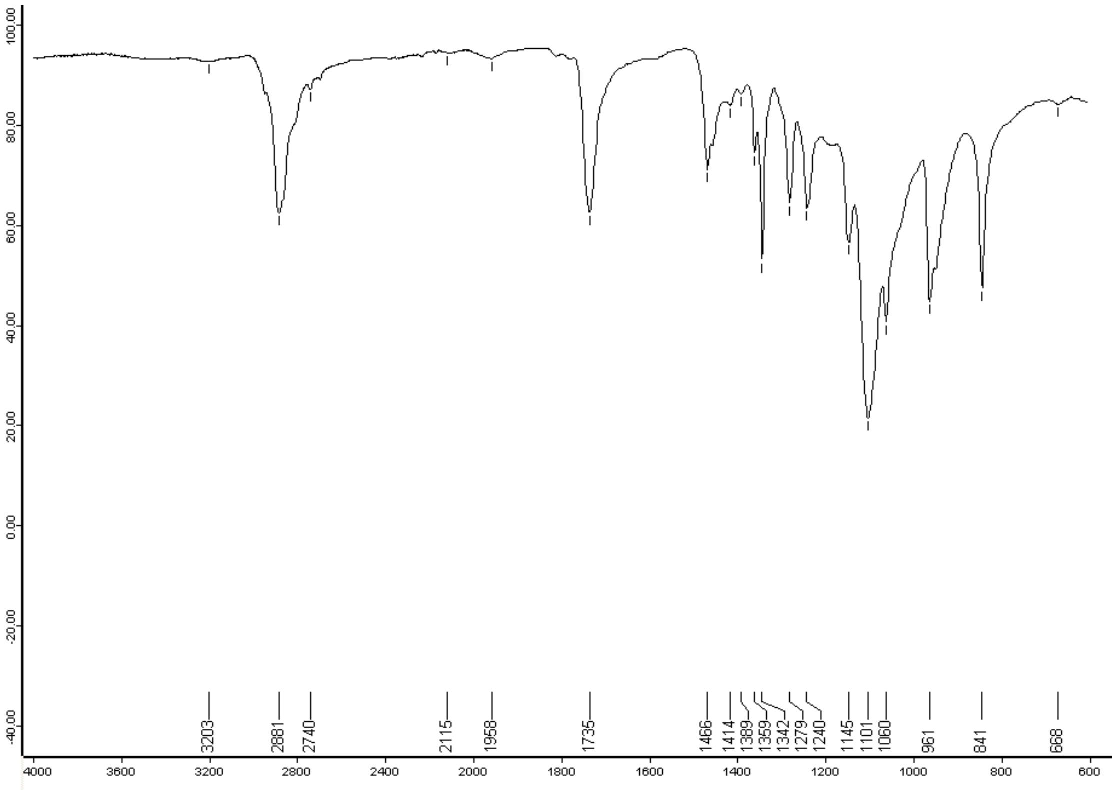

IR (ATR)

^{1}H NMR and IR spectra of oligomer **P2**

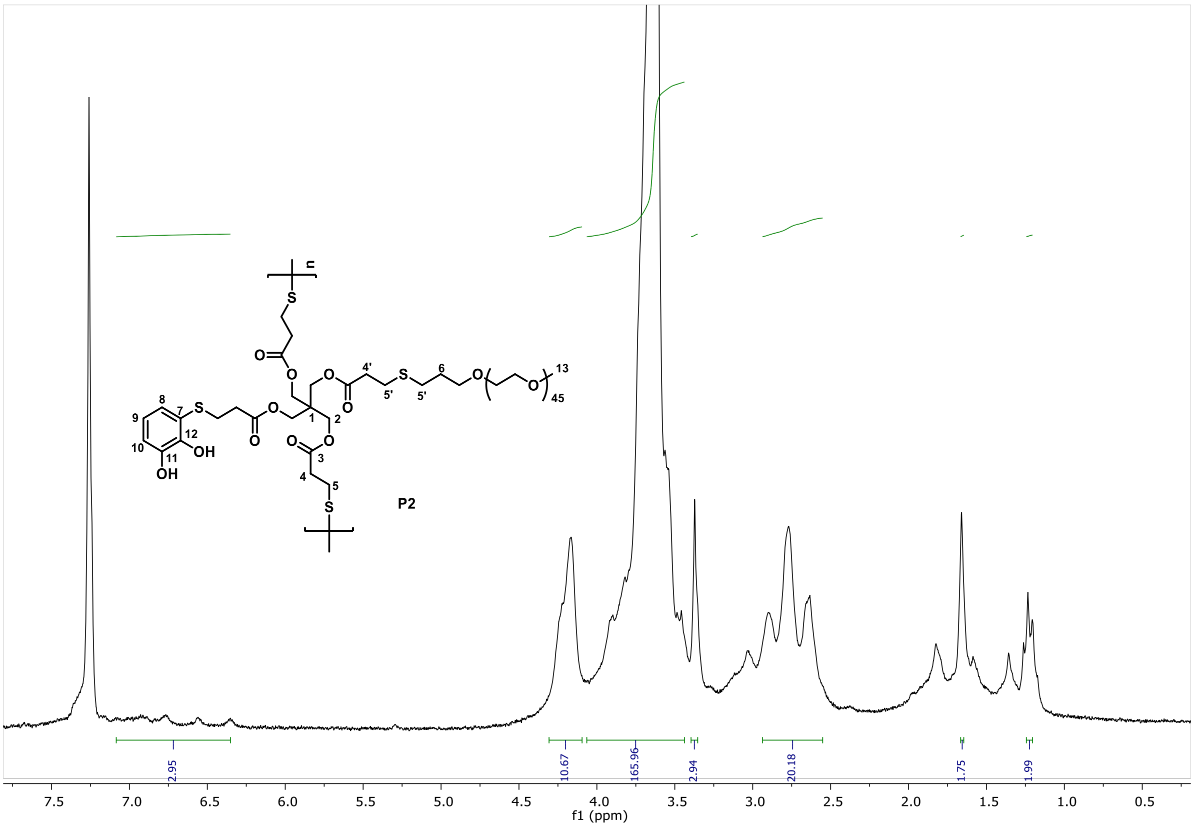

^{1}H NMR (360 MHz, $CDCl_3$)

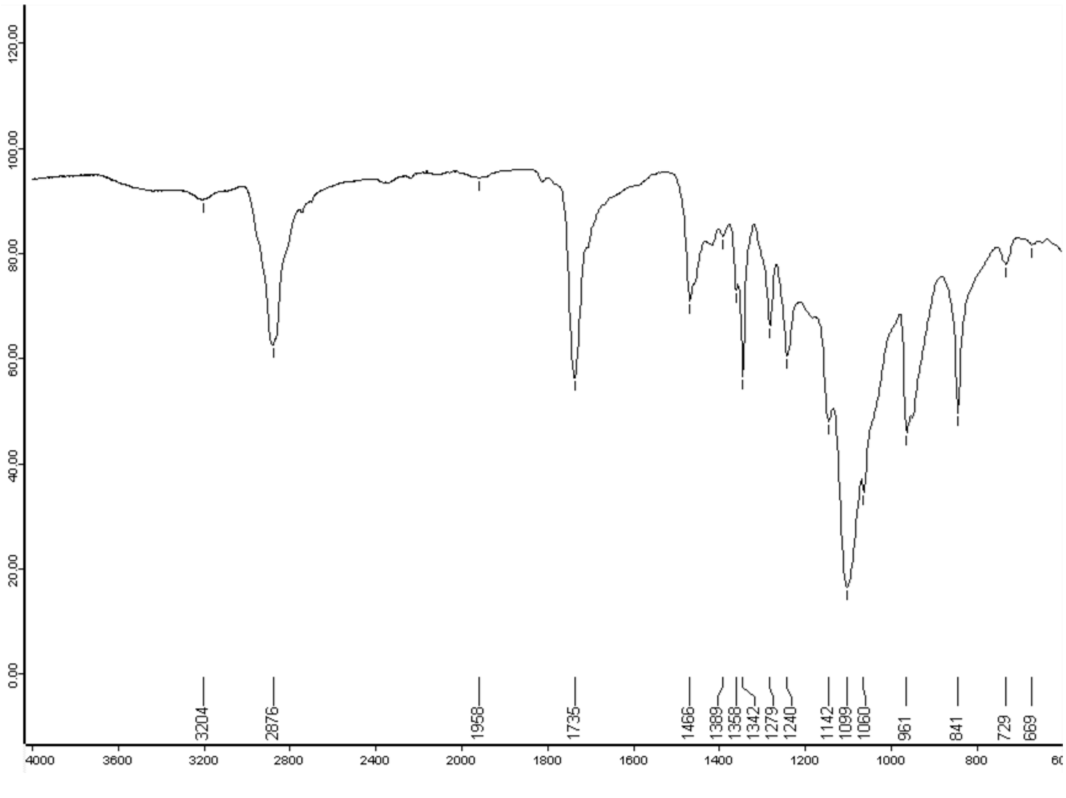

IR (ATR)

^{1}H NMR and IR spectra of oligomer **P5**

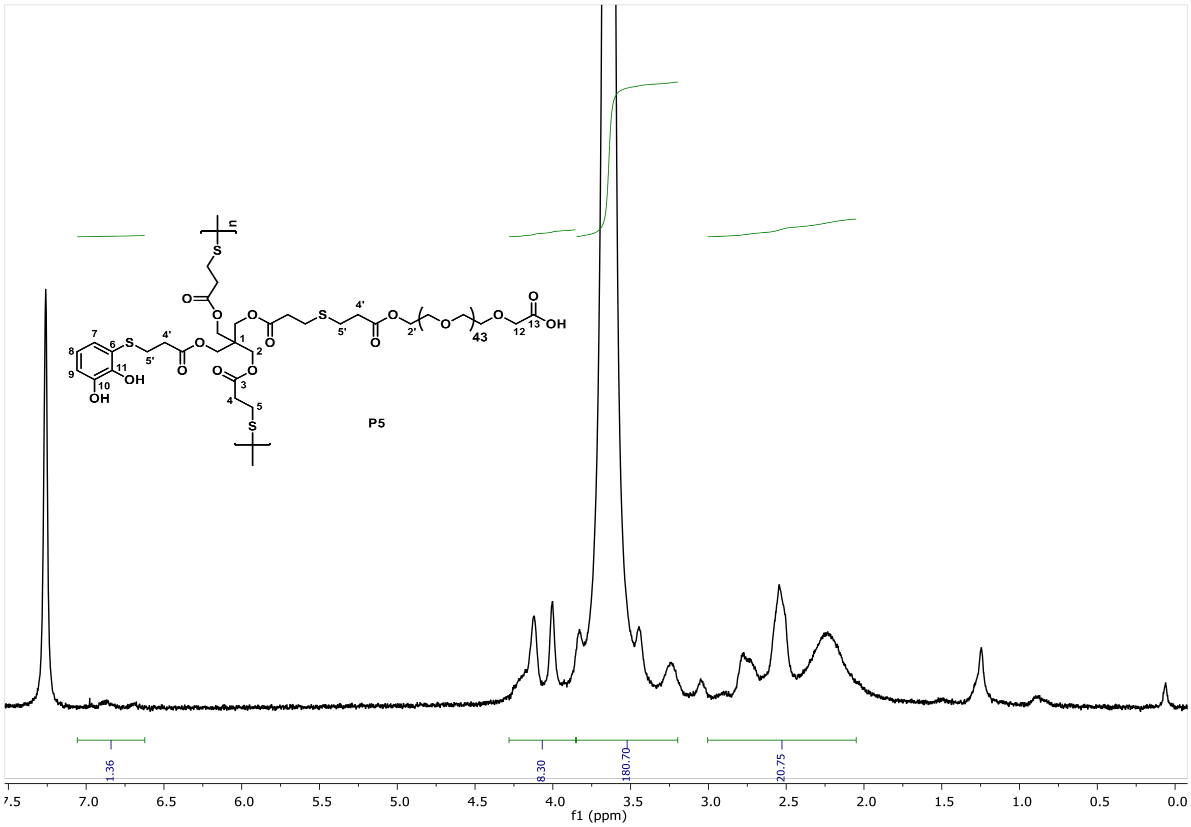

^{1}H NMR (360 MHz, $CDCl_3$)

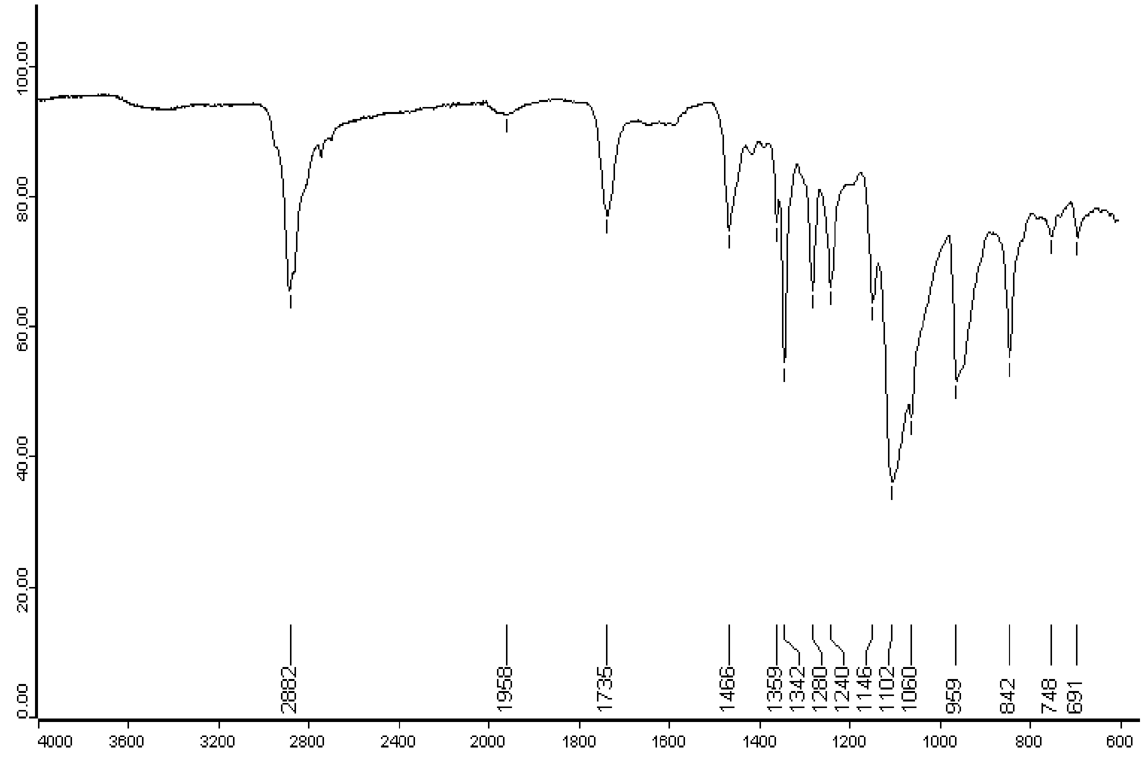

IR (ATR)

^{1}H NMR spectrum of copolimer **C2-3**

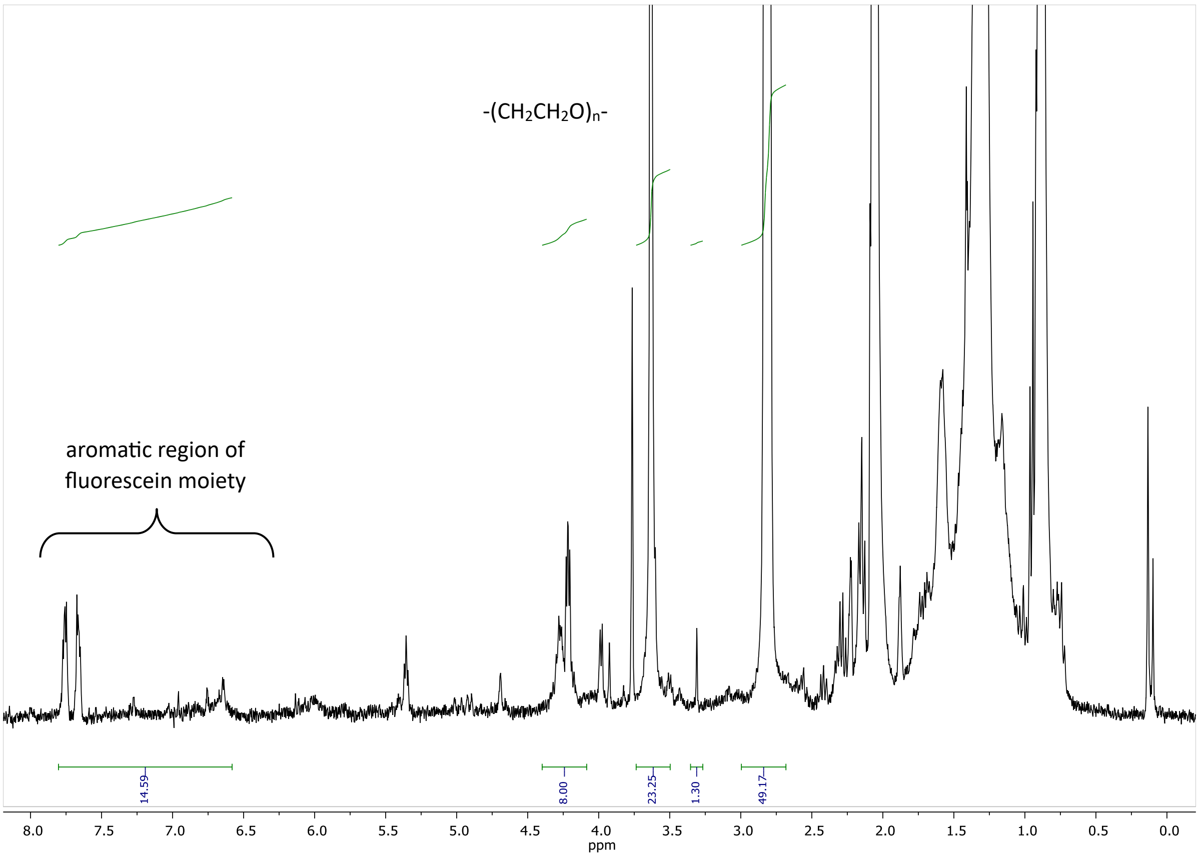

^{1}H NMR (360 MHz, $(CD_3)_2CO$)

S6. GPC spectra

GPC spectra with both IR (top) and UV (bottom) detectors of building block **10**

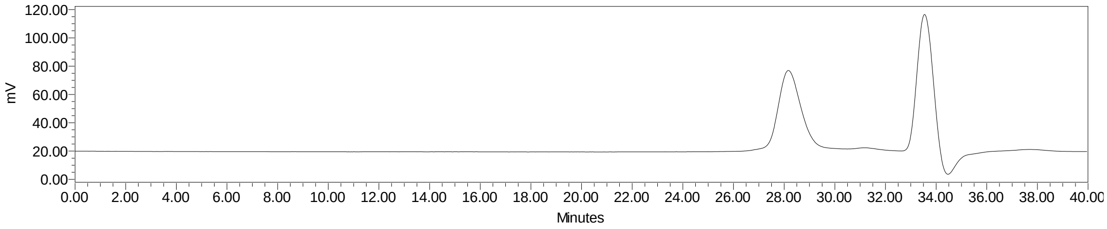

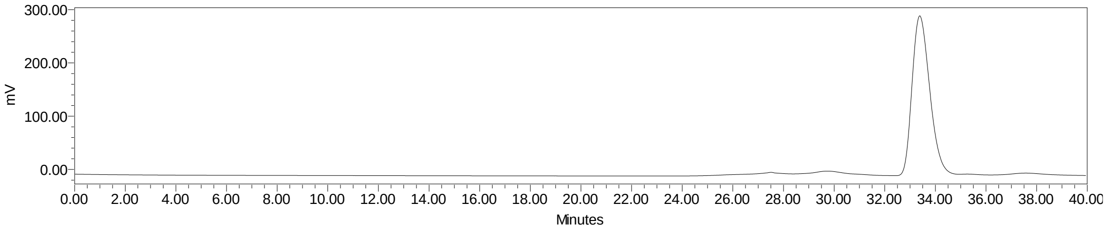

GPC spectra with both IR (top) and UV (bottom) detectors of **P2**

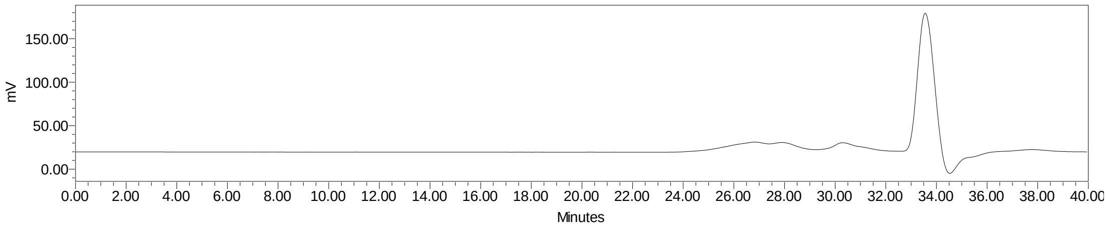

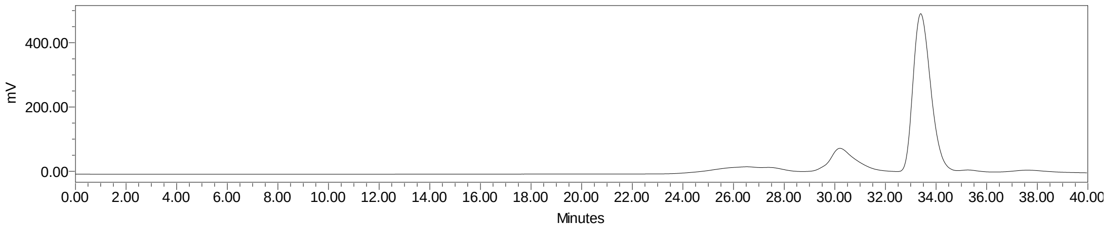

S7. ^{1}H NMR spectra organic part of degraded Fe_3O_4@P5-Amides NPs

^{1}H NMR spectra of the organic part of degraded Fe_3O_4@**P5-Allylamide** NPs

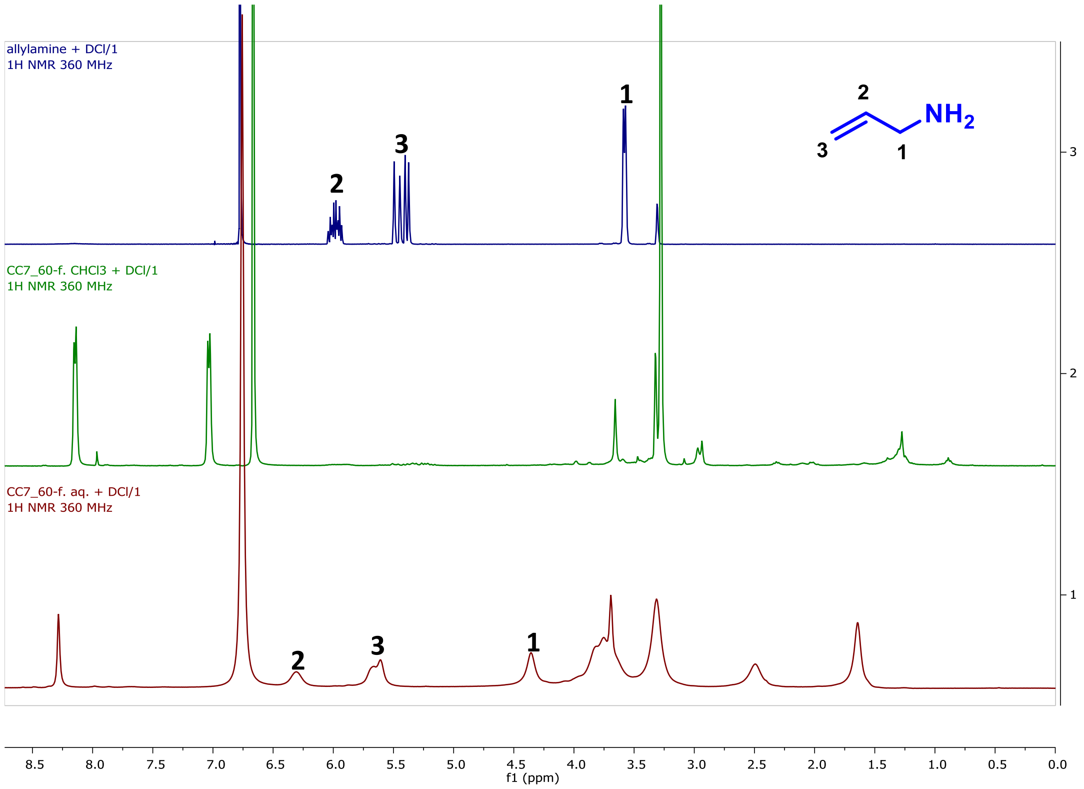

^{1}H NMR spectra from the commercial allylamine (in blue), the residue obtained in the $CHCl_3$ phase once the MNPs have been transferred in the aqueous one (in green), and the MNPs@cat-PEG-allylamide transferred into the aqueous phase (in red). All the ^{1}H NMRs were performed in CD_3OD and all the samples were treated with five drops of DCl and let overnight.

^{1}H NMR spectra of the organic part of degraded Fe_3O_4@**P5-ProtectedSugar amide** NPs

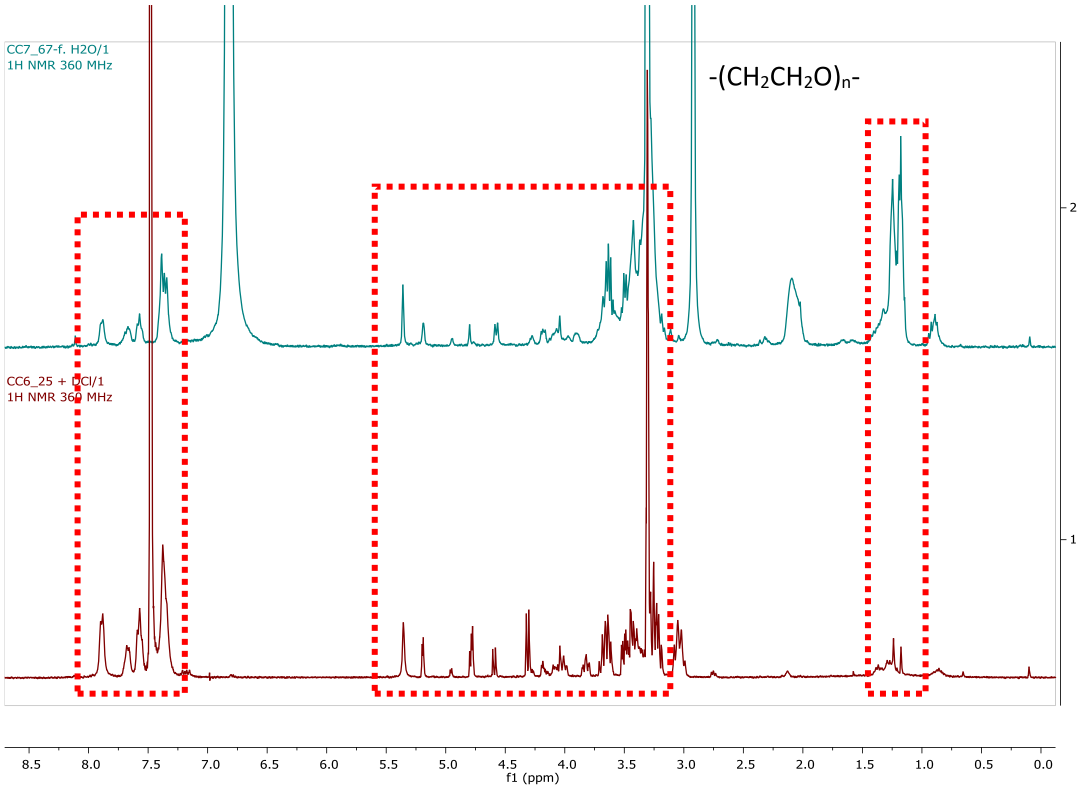

^{1}H NMR spectra from the MNPs@cat-PEG-Protectedsugar amide transferred into the aqueous phase (in blue), and the starting amino-glucofuranose derivative **12** (in red). Both ^{1}H NMRs were performed in CD_3OD and the two samples were previously treated with five drops of DCl overweekend. Peaks corresponding to the amino sugar are dashed framed in red, and the peak at 2.92 ppm in the above spectrum corresponds to the PEG chain.

^{13}C NMR spectra of the organic part of degraded Fe_3O_4@**P5-Sugar amide** NPs

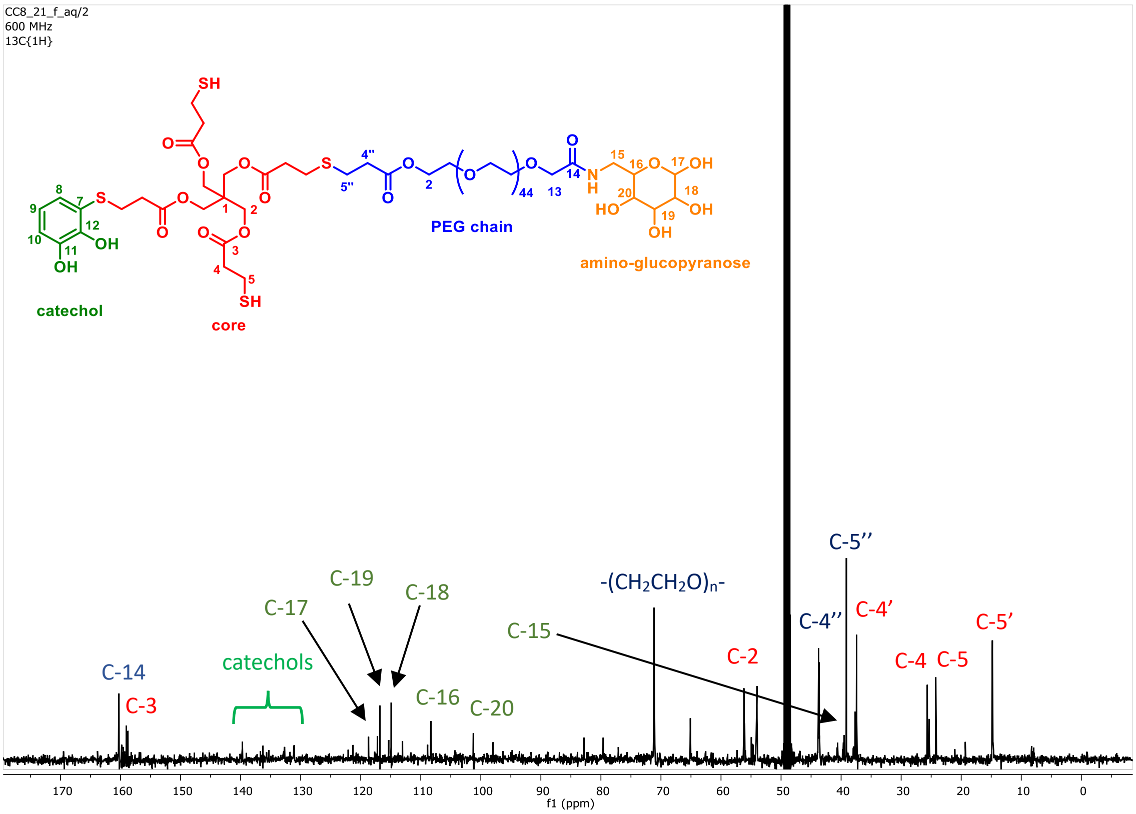

^{13}C NMR spectrum in CD_3OD of MNP@cat-PEG-amino-glucopyranose treated with five drops of DCl (35% wt in D_2O). In green signals corresponding to the catechol moiety; in red signals corresponding to the pentaerythritol tetrakis(3-mercaptopropionate); in blue signals corresponding to the PEG chain; and in orange signals corresponding to the amino-glucopyranose derivative.
